# Author Correction: Genome-wide translational profiling of amygdala *Crh*-expressing neurons reveals role for CREB in fear extinction learning

**DOI:** 10.1038/s41467-020-19534-x

**Published:** 2020-10-27

**Authors:** Kenneth M. McCullough, Chris Chatzinakos, Jakob Hartmann, Galen Missig, Rachael L. Neve, Robert J. Fenster, William A. Carlezon, Nikolaos P. Daskalakis, Kerry J. Ressler

**Affiliations:** 1 McLean Hospital, Department of Psychiatry, Harvard Medical School, Belmont, MA 02478 USA; 2 Gene Transfer Core, Massachusetts General Hospital, Boston, MA 02114 USA

**Keywords:** Transcriptomics, Amygdala, Molecular neuroscience

Correction to: *Nature Communications* 10.1038/s41467-020-18985-6, published online 14 October 2020.

The original version of this Article’s Acknowledgment section incorrectly read:

This work was supported by funding from Cohen Veteran Biosciences, NIMH (P50-MH115874, R01-MH108665, and R01-MH117292), and the Frazier Institute at McLean Hospital. C.C. was supported by the 2019 Seed Grant (through NIMH P50-MH115874). N.P.D. was supported by a NARSAD Young Investigator grant and an appointed KL2 award from Harvard Catalyst/The Harvard Clinical and Translational Science Center (NCATS KL2TR002542 and UL1TR002541).

The correct version reads:

This work was supported by funding from Cohen Veterans Bioscience, NIMH (P50-MH115874, R01-MH108665, R01-MH117292, and R01-MH063266), and the Frazier Institute at McLean Hospital. C.C. was supported by the 2019 SPARED Center Seed Grant (through NIMH P50-MH115874). G.M. was supported by a NARSAD Young Investigator grant. N.P.D. was supported by a NARSAD Young Investigator grant and an appointed KL2 award from Harvard Catalyst/The Harvard Clinical and Translational Science Center (NCATS KL2TR002542 and UL1TR002541).

This has now been corrected in both the PDF and HTML versions of the Article.

