# Supplementary material for: Genome-wide translational profiling of amygdala Crh-expressing neurons reveals role for CREB in fear extinction learning
Source: Nat Commun. 2020 Oct 14;11:5180. doi: 10.1038/s41467-020-18985-6 (PMC7560654; doi:10.1038/s41467-020-18985-6)
Supplement: Supplementary file 1 — Supplementary Information [file 41467_2020_18985_MOESM1_ESM.pdf]

Supplementary Information for

Genome-wide translational profiling of amygdala *Crh*-expressing neurons reveals role for CREB in fear extinction learning

Kenneth M. McCullough[1], Christos Chatzinakos[1], Jakob Hartmann[1],

Galen Missig[1], Rachael L. Neve[2], Robert J. Fenster[1], William A. Carlezon Jr.[1],

Nikolaos P. Daskalakis[1, 3, ✉], and Kerry J. Ressler[1, 3, ✉]

Affiliations:

[1]McLean Hospital, Harvard Medical School, Belmont, MA 02478, USA.

[2]Gene Transfer Core, Massachusetts General Hospital, Boston, MA 02114, USA.

[3]These authors contributed equally.

✉email: ndaskalakis@mclean.harvard.edu; kressler@mclean.harvard.edu

Document includes 5 supplementary figures.

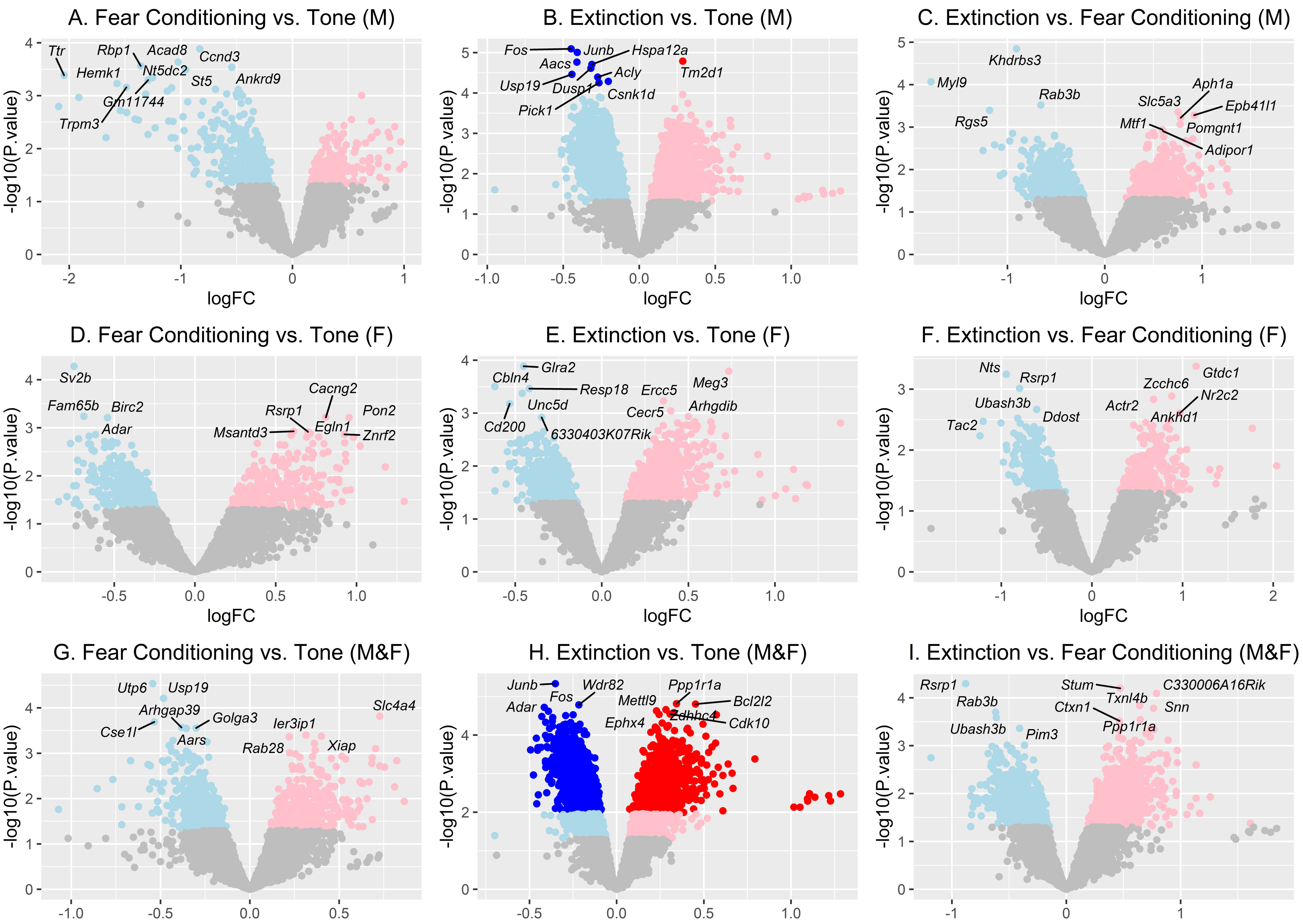

Supplementary Figure 1

Supplementary Figure 1. Differential gene expression analysis

Volcano plots (i.e., p-value (in -log10 scale) by fold changes (in log2 scale)) differential expression analysis identified differentially expressed genes (DEG) according to the three pairwise comparisons: Fear Conditioning (FC) vs. Tone Alone (TA), Extinction (EXT) vs. TA, and EXT vs. FC in male (A-C), female (D-F), and combined male and female (G-I). Analysis was performed using *limma* with Benjamini Hochberg's False Discovery Rate (FDR) multiple testing correction of p-values. Red dots indicate upregulated genes, while blue indicate downregulated genes. When the colors are more intense this indicates FDR-adjusted p-value < 0.05. FDR p-value cut-offs are calculated as a function of the p-value distribution within each analysis and, thus, are specific to each comparison.

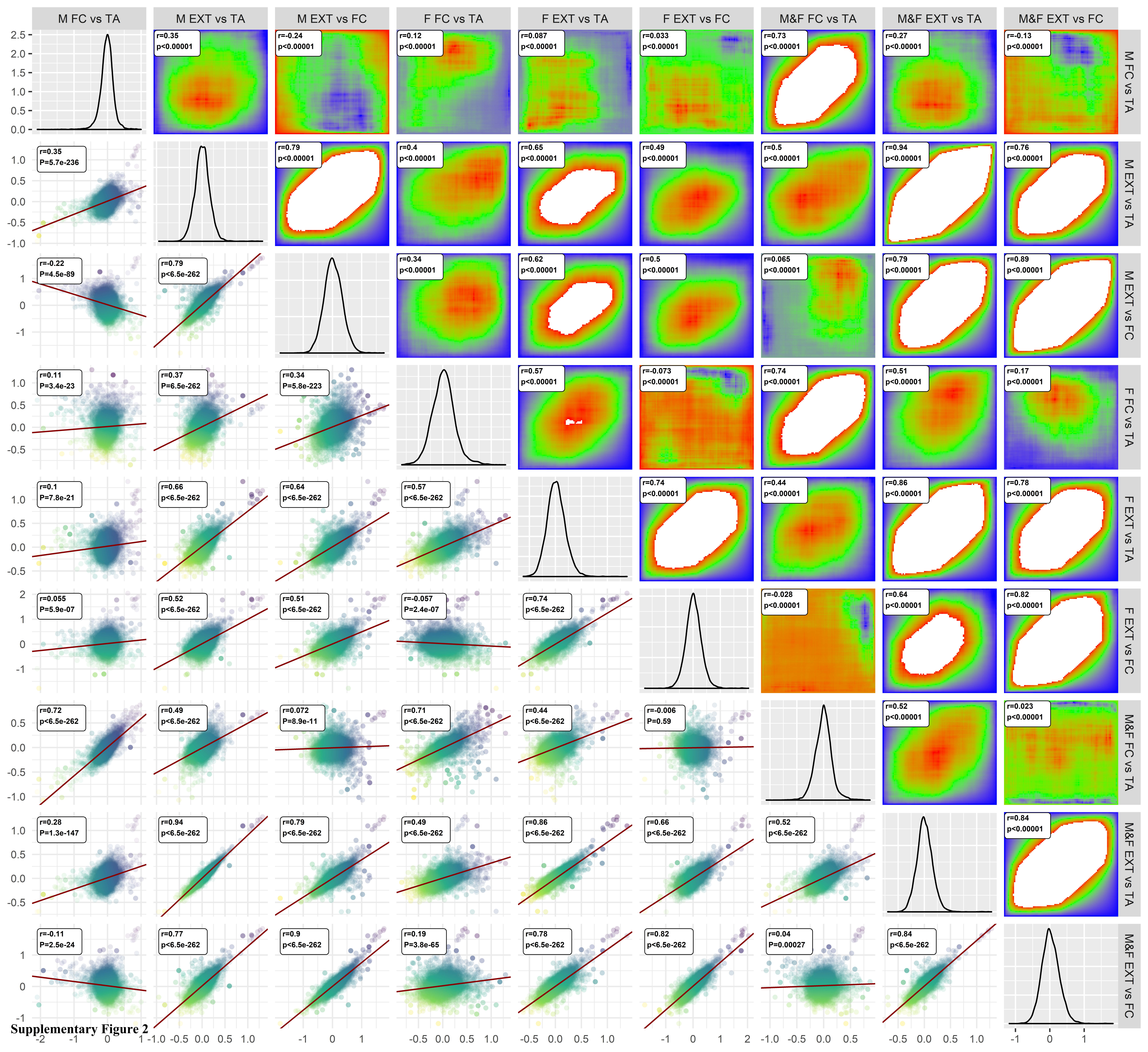

Supplementary Figure 2

Supplementary Figure 2. **Correlation analysis of differential gene expression results.** Spearman Correlation (down triangle) of fold-changes (log2FC) and rank-rank hypergeometric overlap (upper triangle) of direction-signed p-values from the differential expression analyses based on between the three groups: Tone Alone (TA), Conditioning (FC) and Extinction (EXT) in both sexes: males (M) and females (F) and in across sexes (M&F).

Supplementary Figure 3a

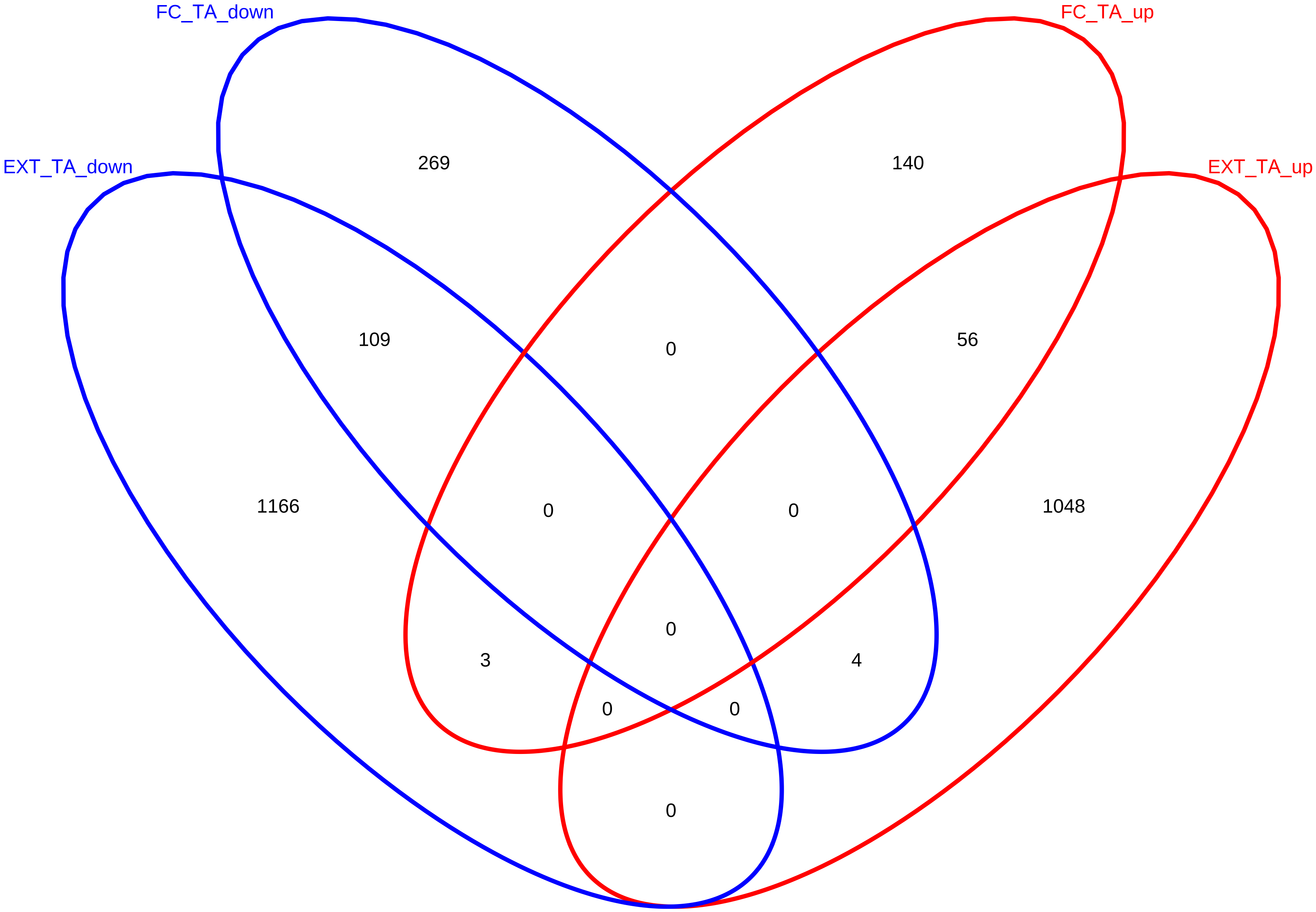

Supplementary Figure 3b

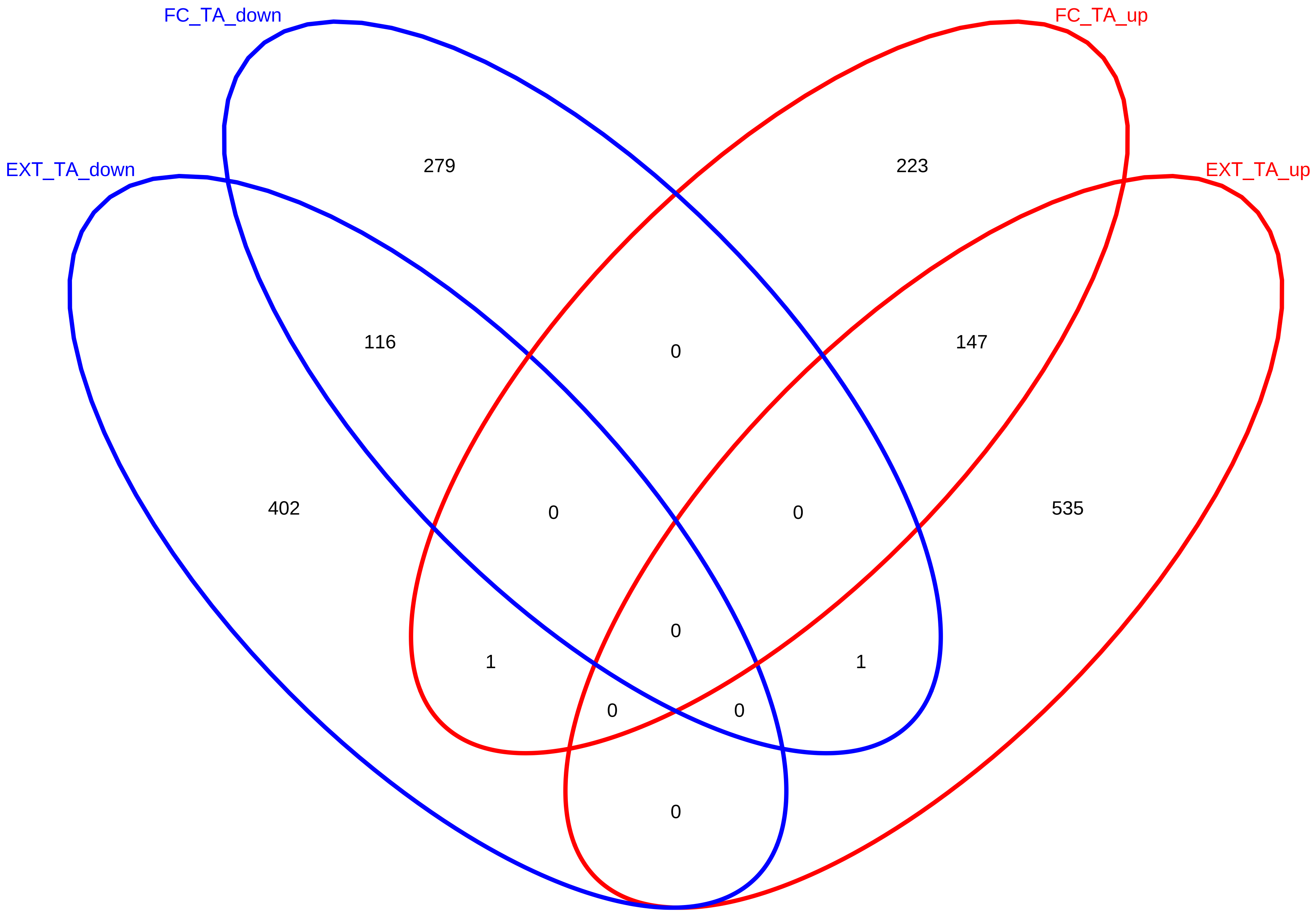

Supplementary Figure 3. Overlap in FC- and EXT- associated genes in males and females.

a. Venn diagram revealed unique and shared up-(red ellipse) and down-(blue ellipse) regulated genes by FC and EXT for males.

b. Venn diagram revealed unique and shared up-(red ellipse) and down-(blue ellipse) regulated genes by FC and EXT for females.

Supplementary Figure 4a

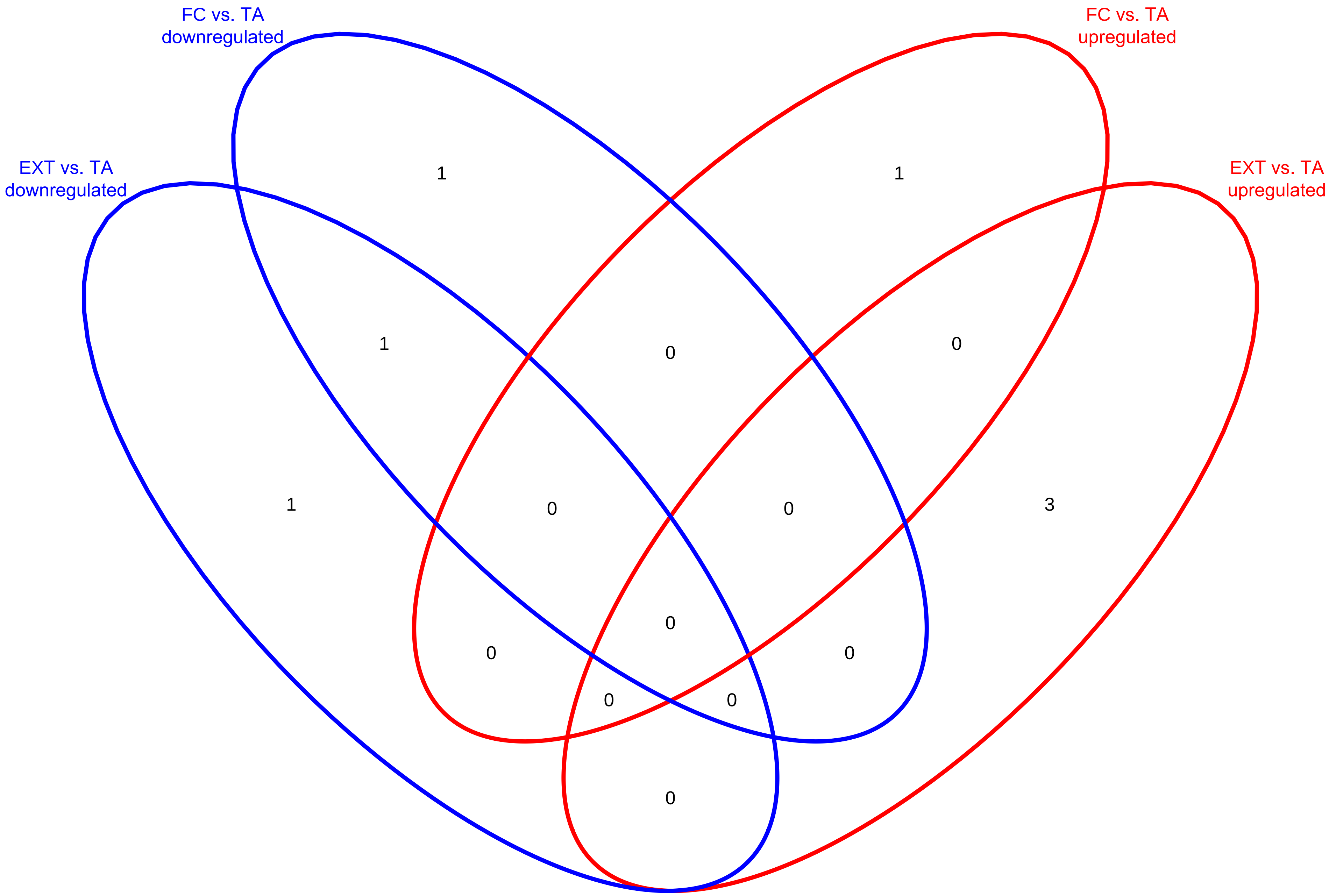

Supplementary Figure 4b

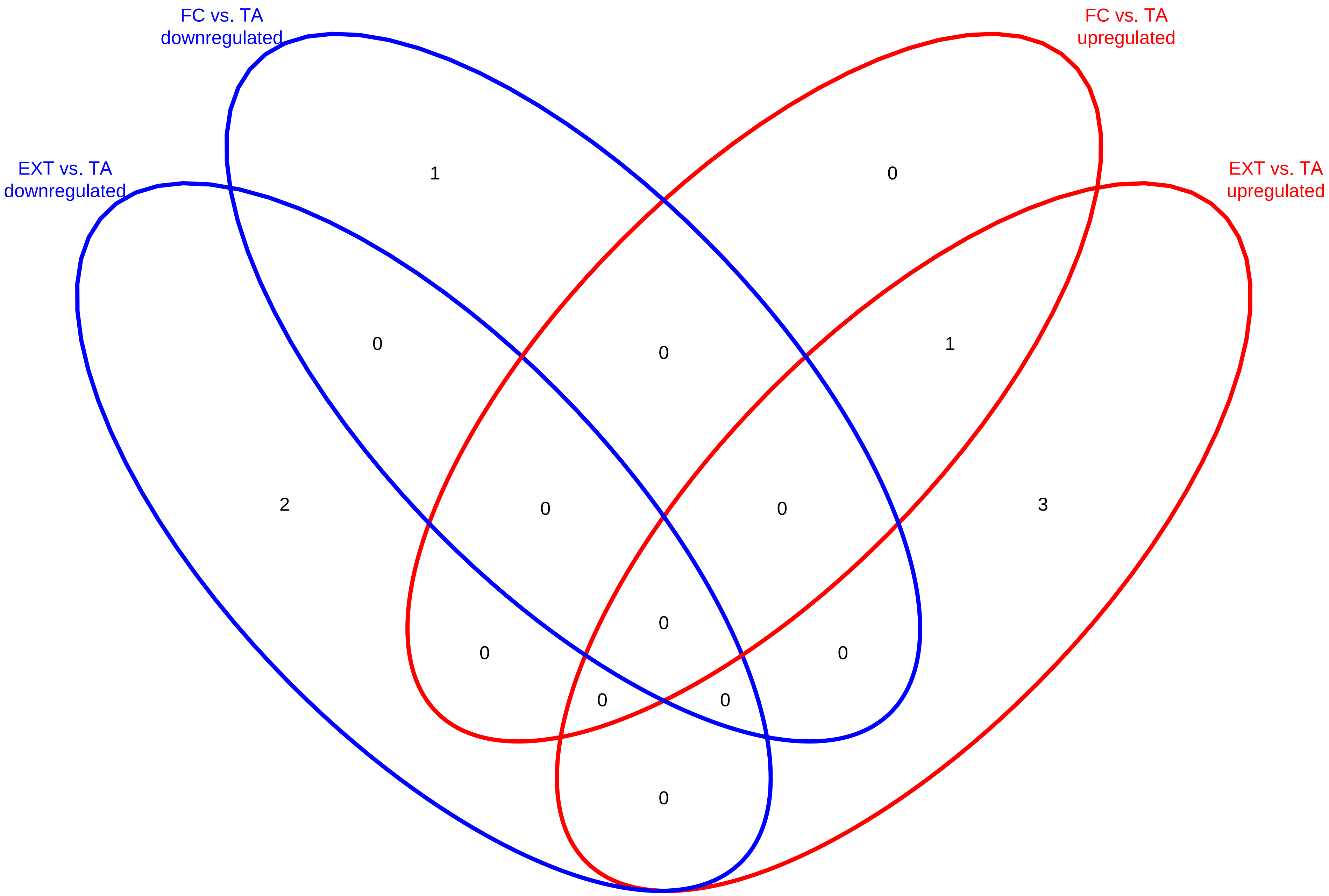

Supplementary Figure 4. Overlap in FC- and EXT- associated gene networks in males and females.

a. Venn diagram revealed unique and shared up-(red ellipse) and down-(blue ellipse) regulated gene networks / modules by Fear Conditioning (FC) and Extinction (EXT) in males.

b. Venn diagram revealed unique and shared up-(blue ellipse) and down-(blue ellipse) regulated gene networks / modules by Fear Conditioning/FC and Extinction/EXT in females.

Supplementary Figure 5a-1

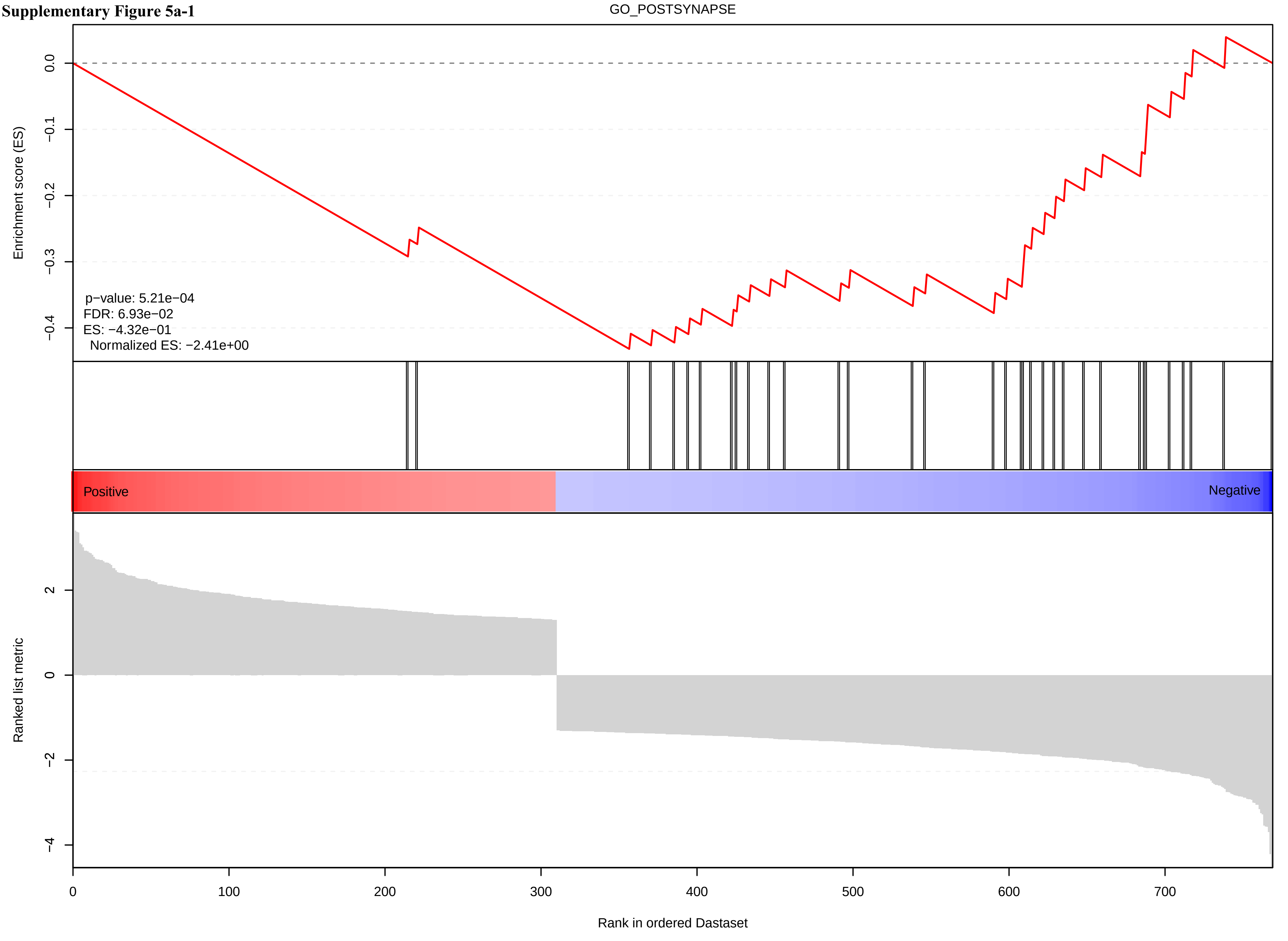

Supplementary Figure 5a-2

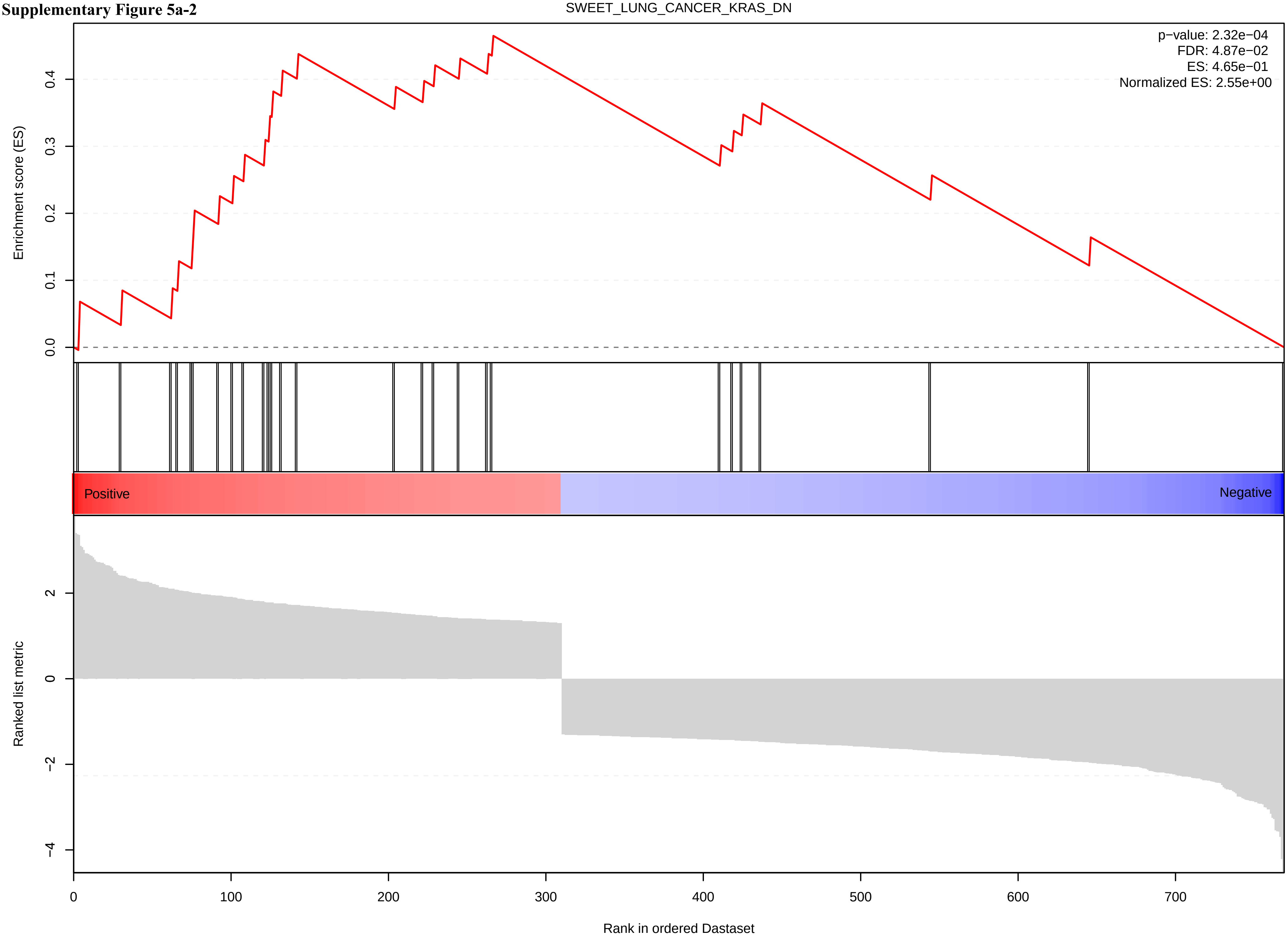

Supplementary Figure 5a-3

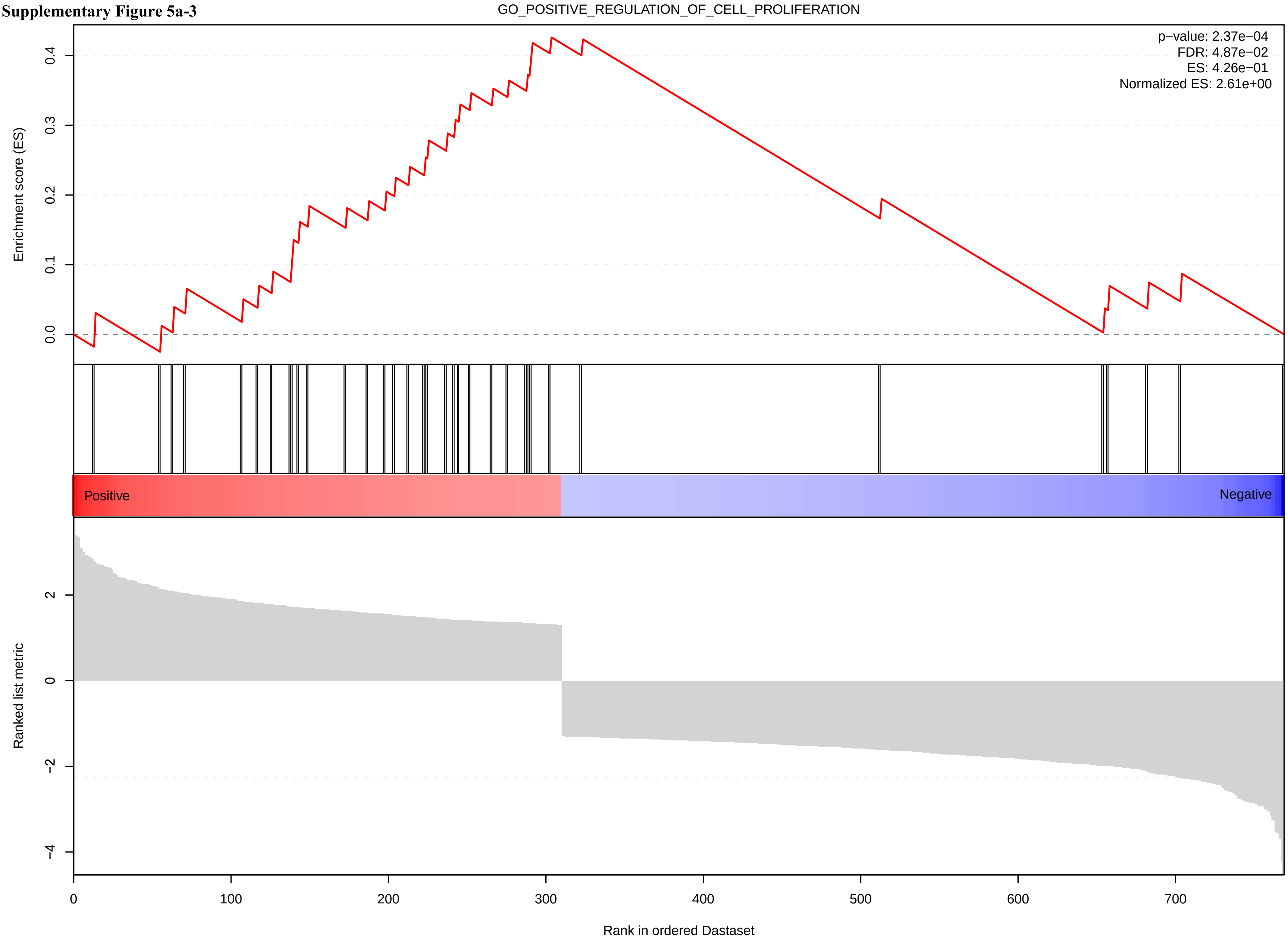

Supplementary Figure 5a-4

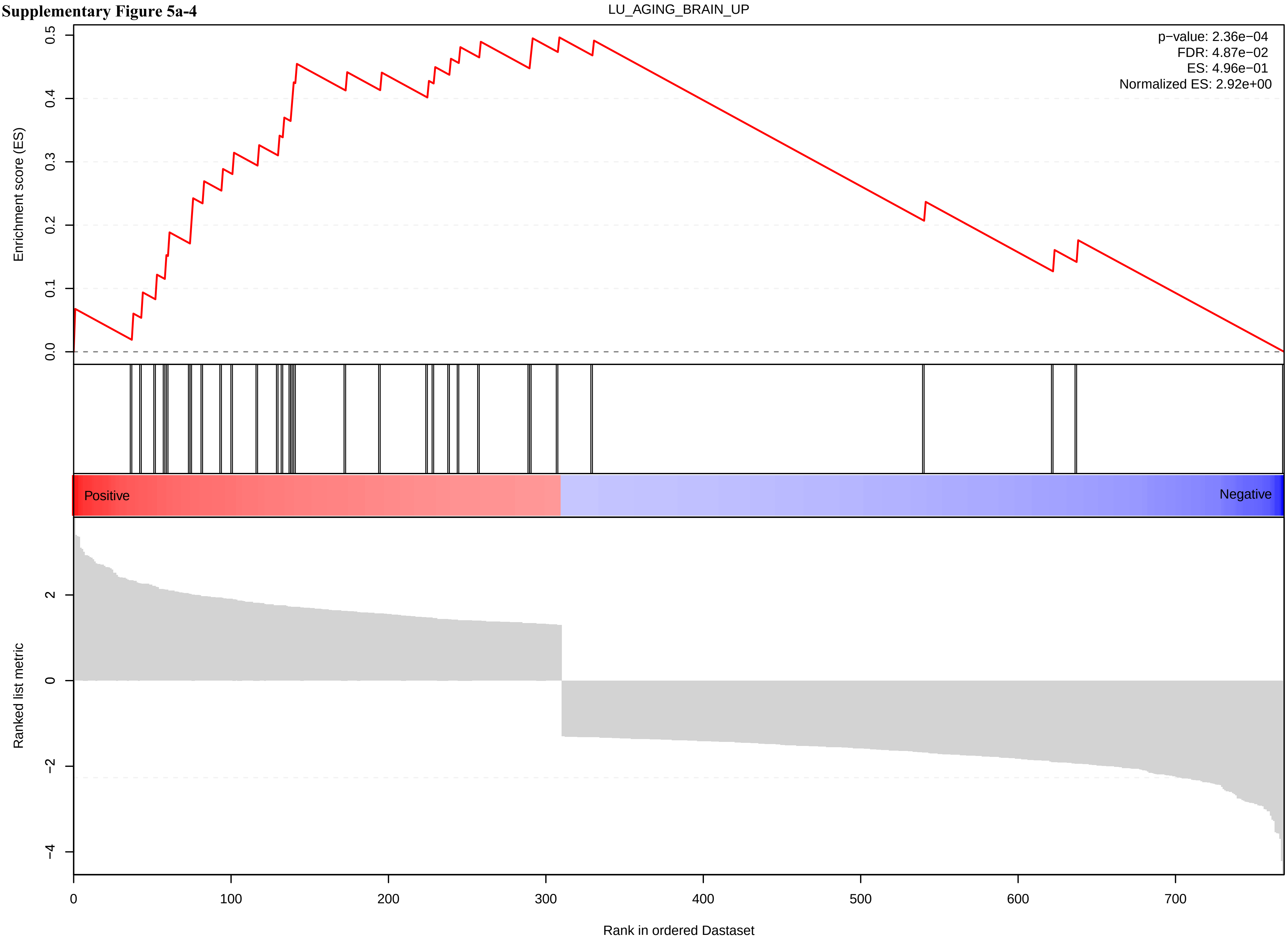

Supplementary Figure 5a-5

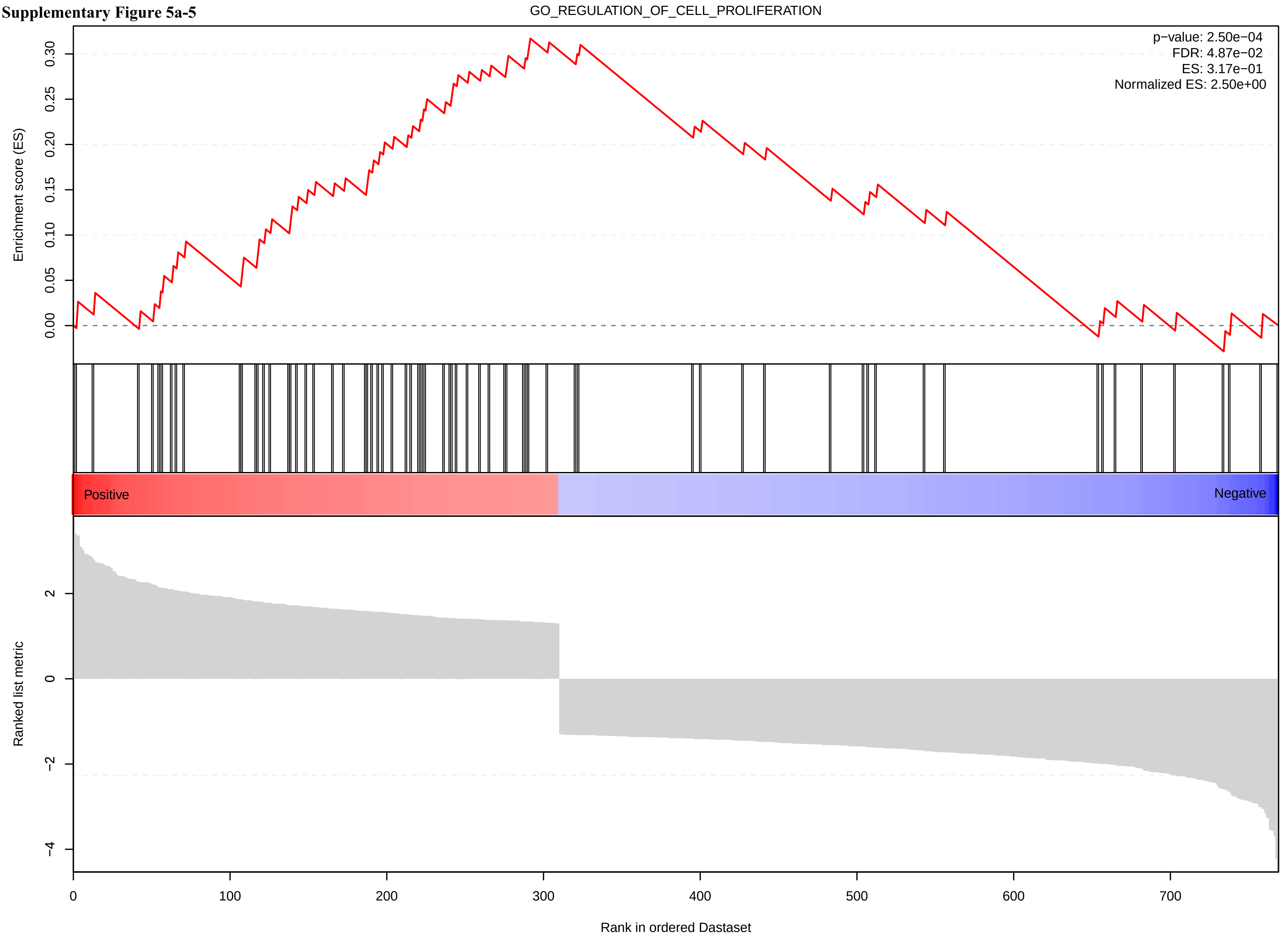

Supplementary Figure 5a-6

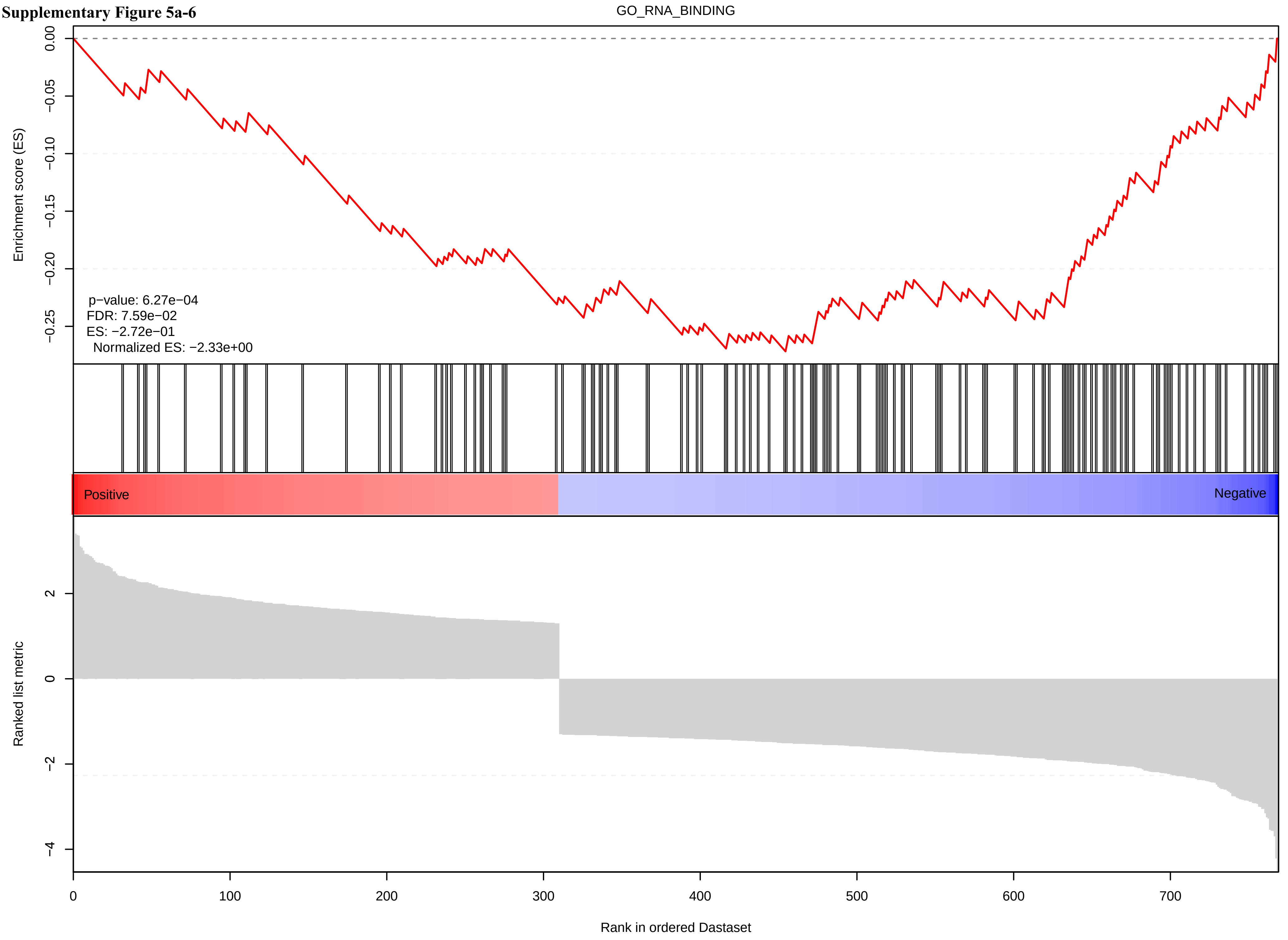

Supplementary Figure 5a-7

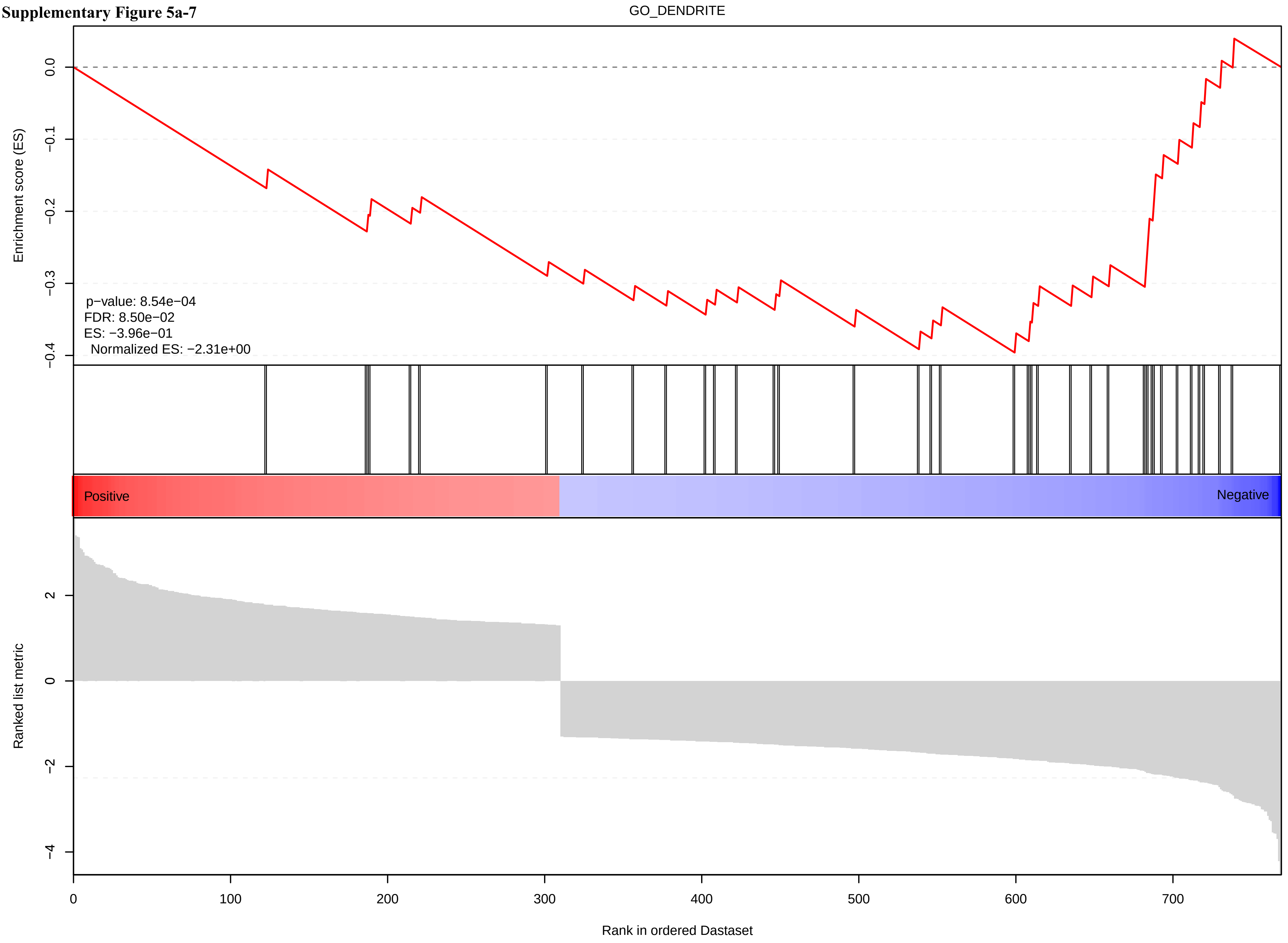

Supplementary Figure 5a-8

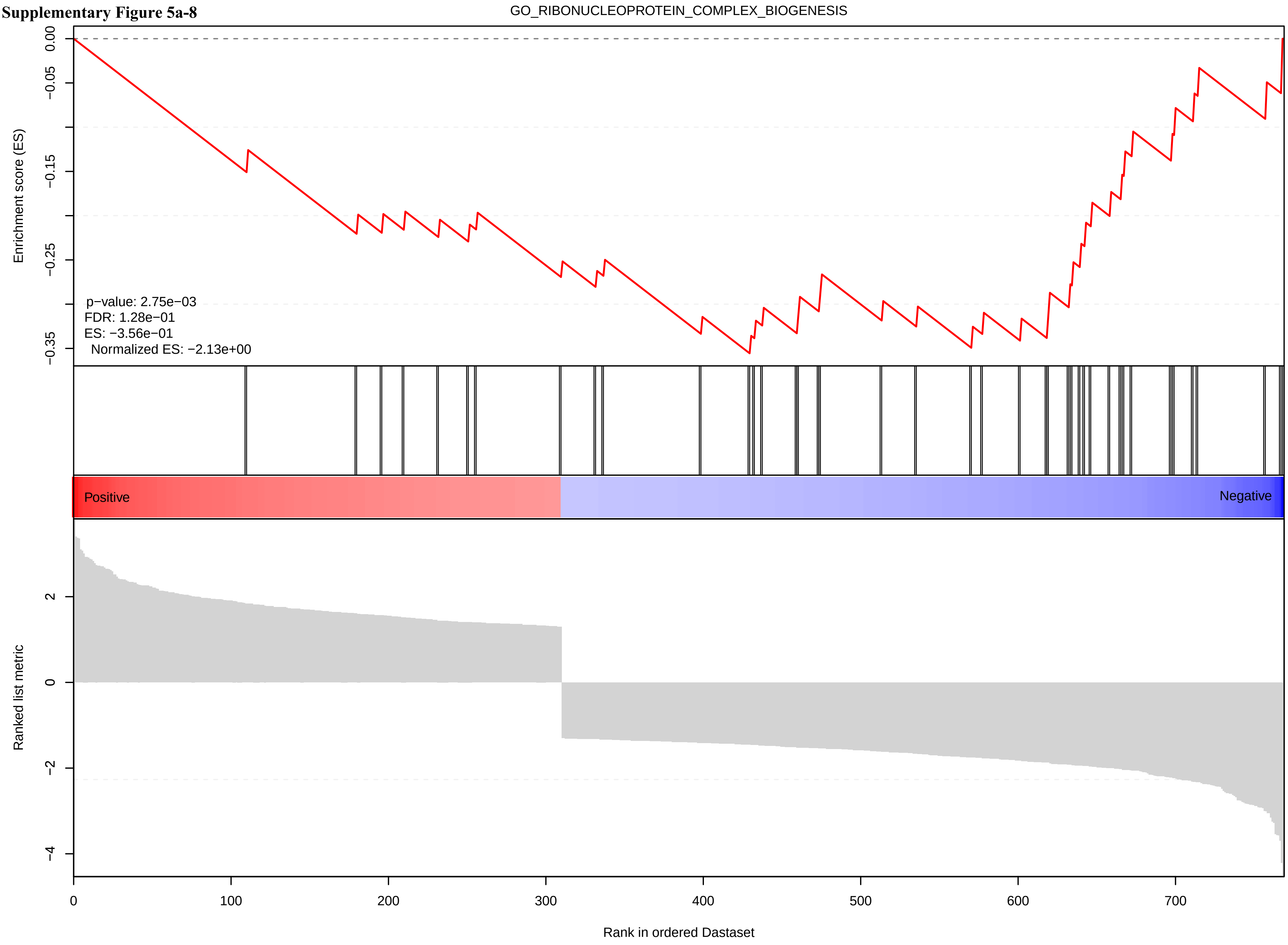

Supplementary Figure 5a-9

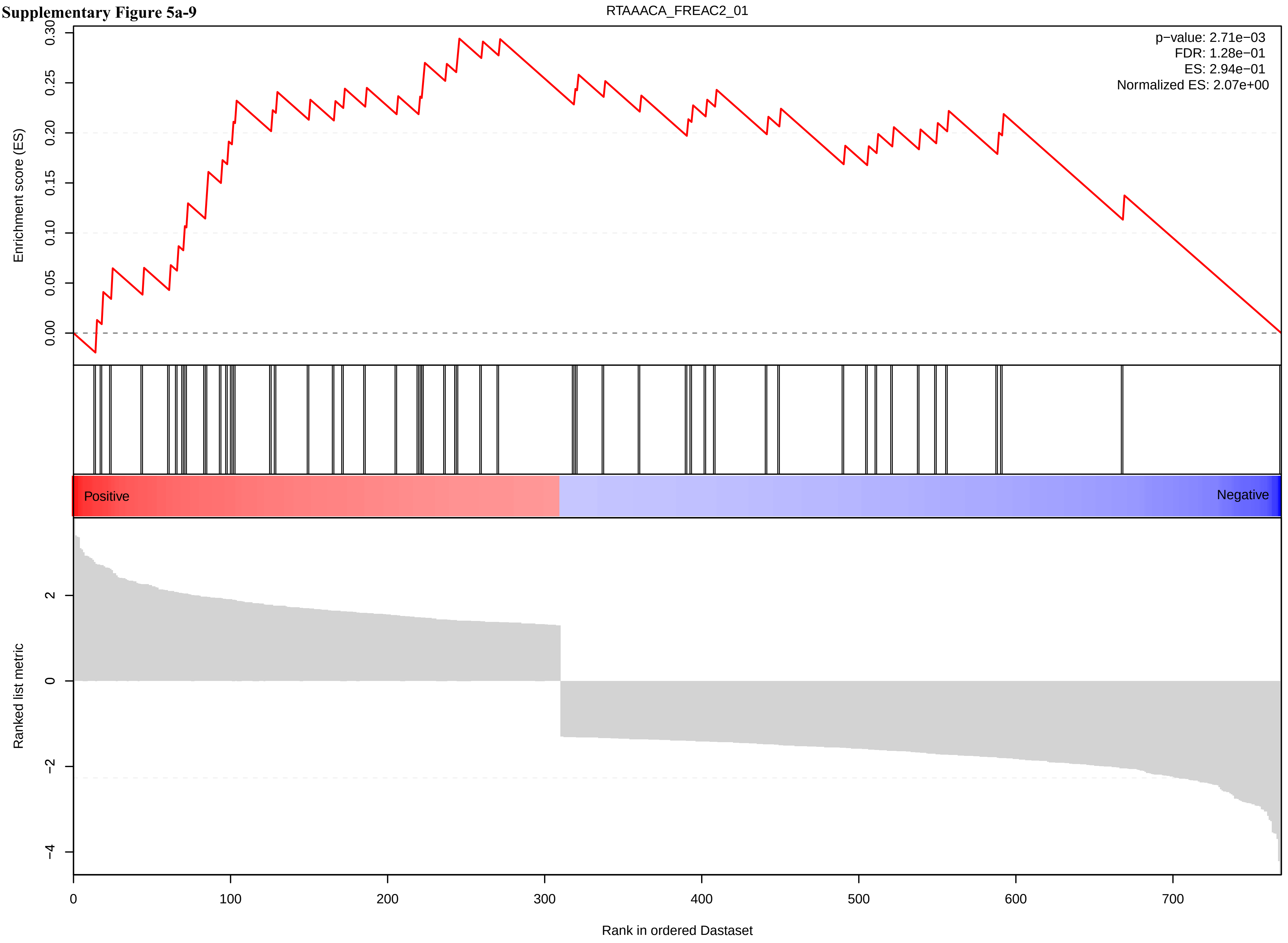

Supplementary Figure 5a-10

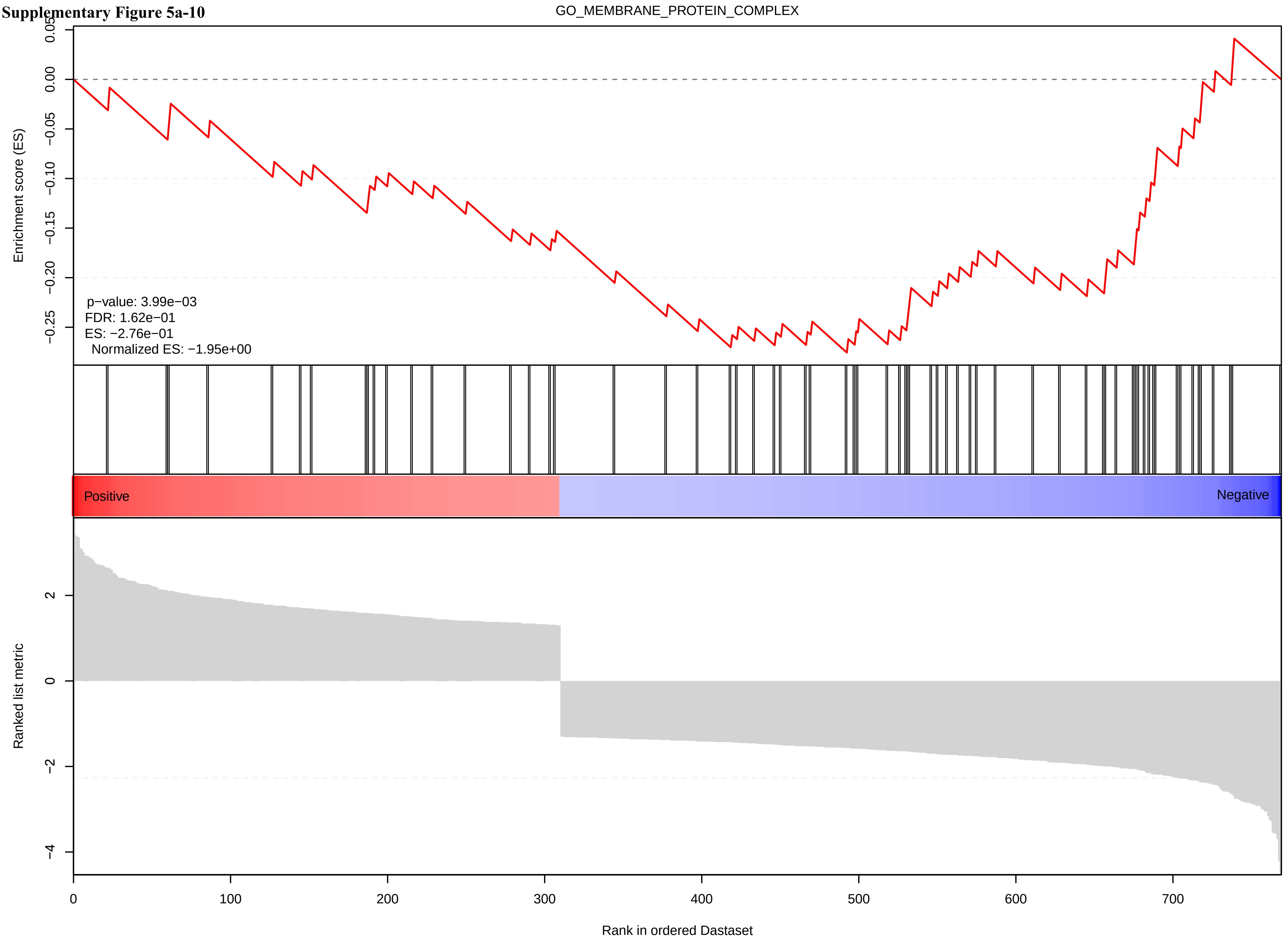

Supplementary Figure 5a-11

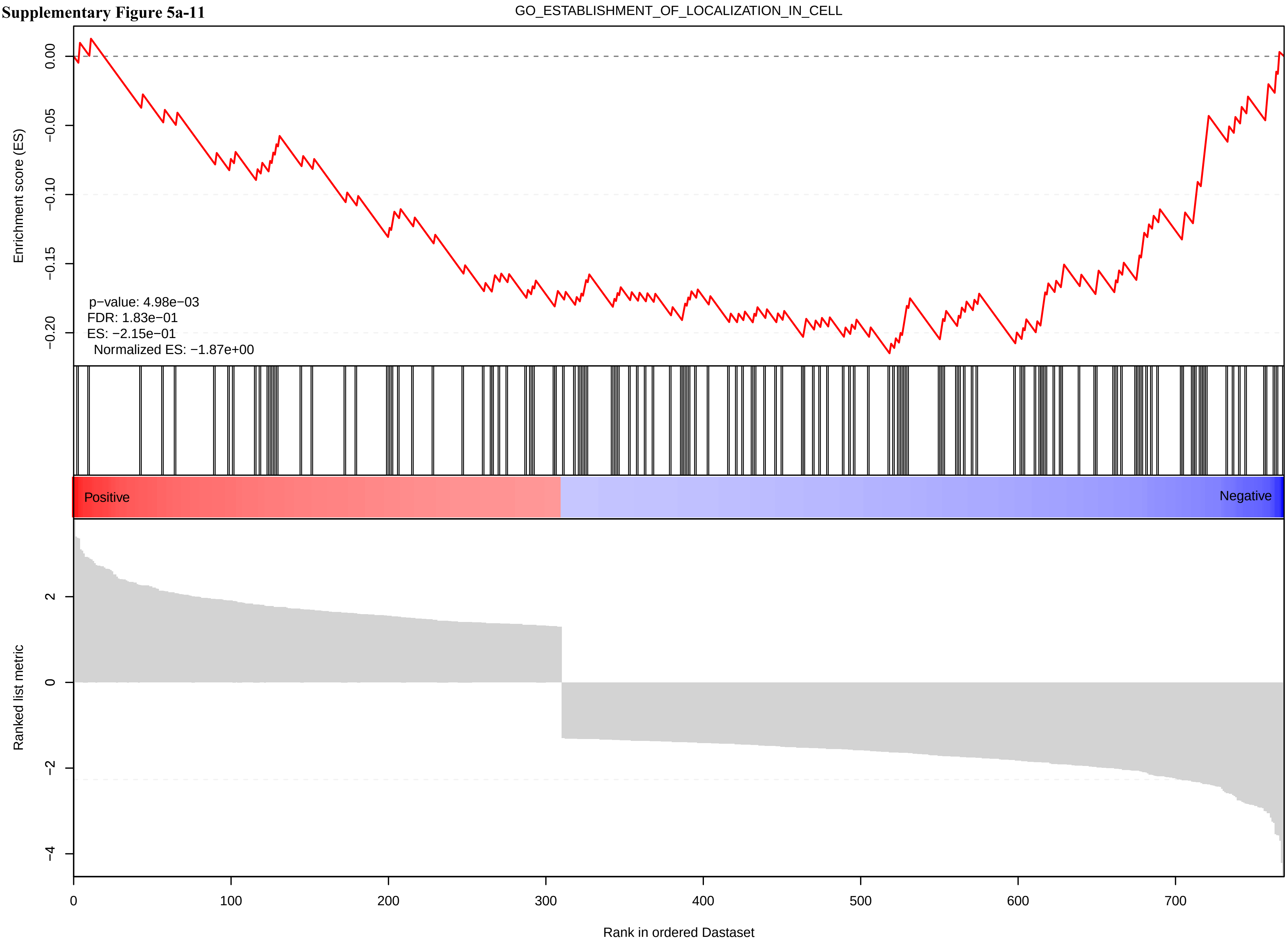

Supplementary Figure 5a-12

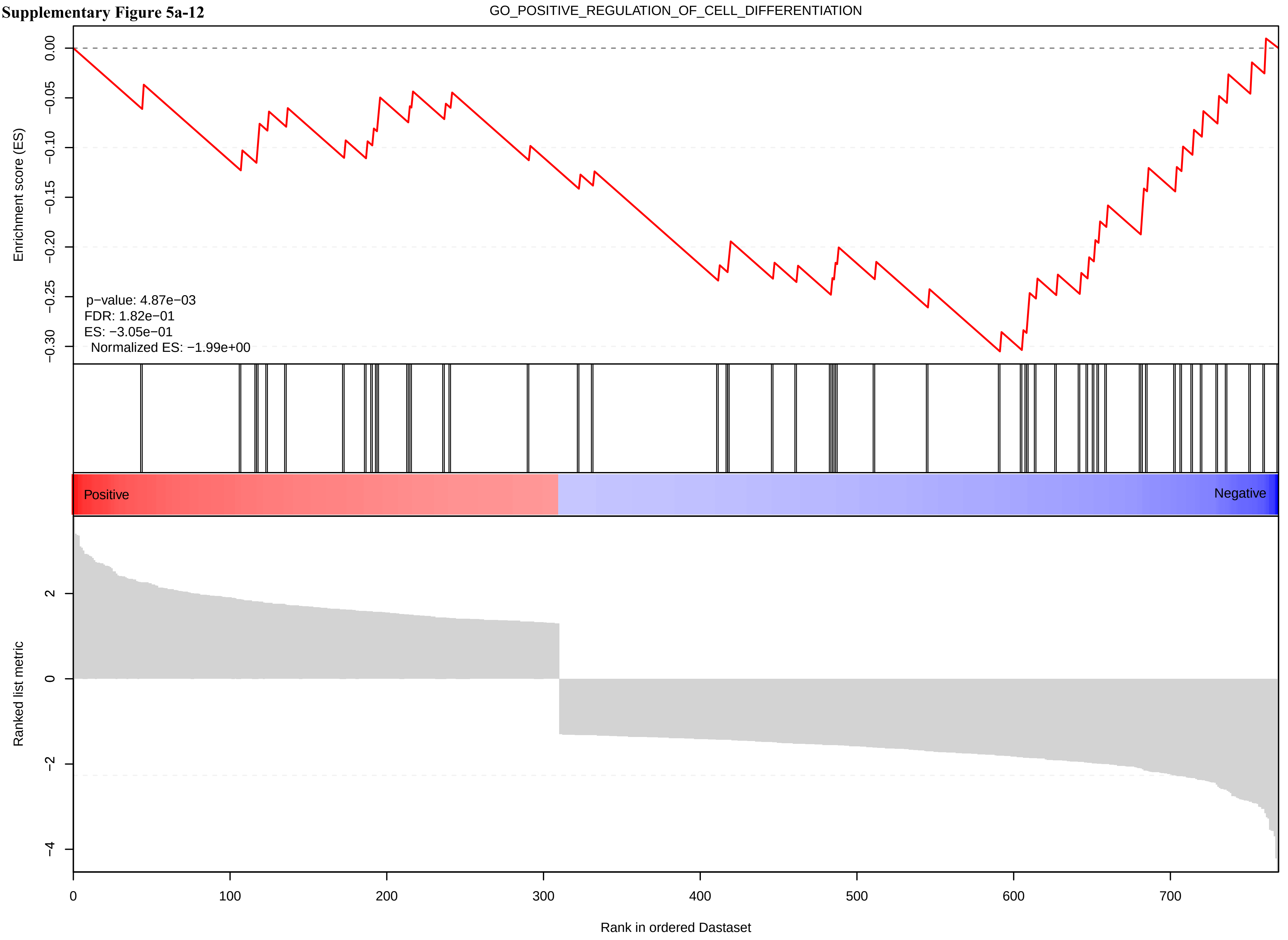

Supplementary Figure 5a-13

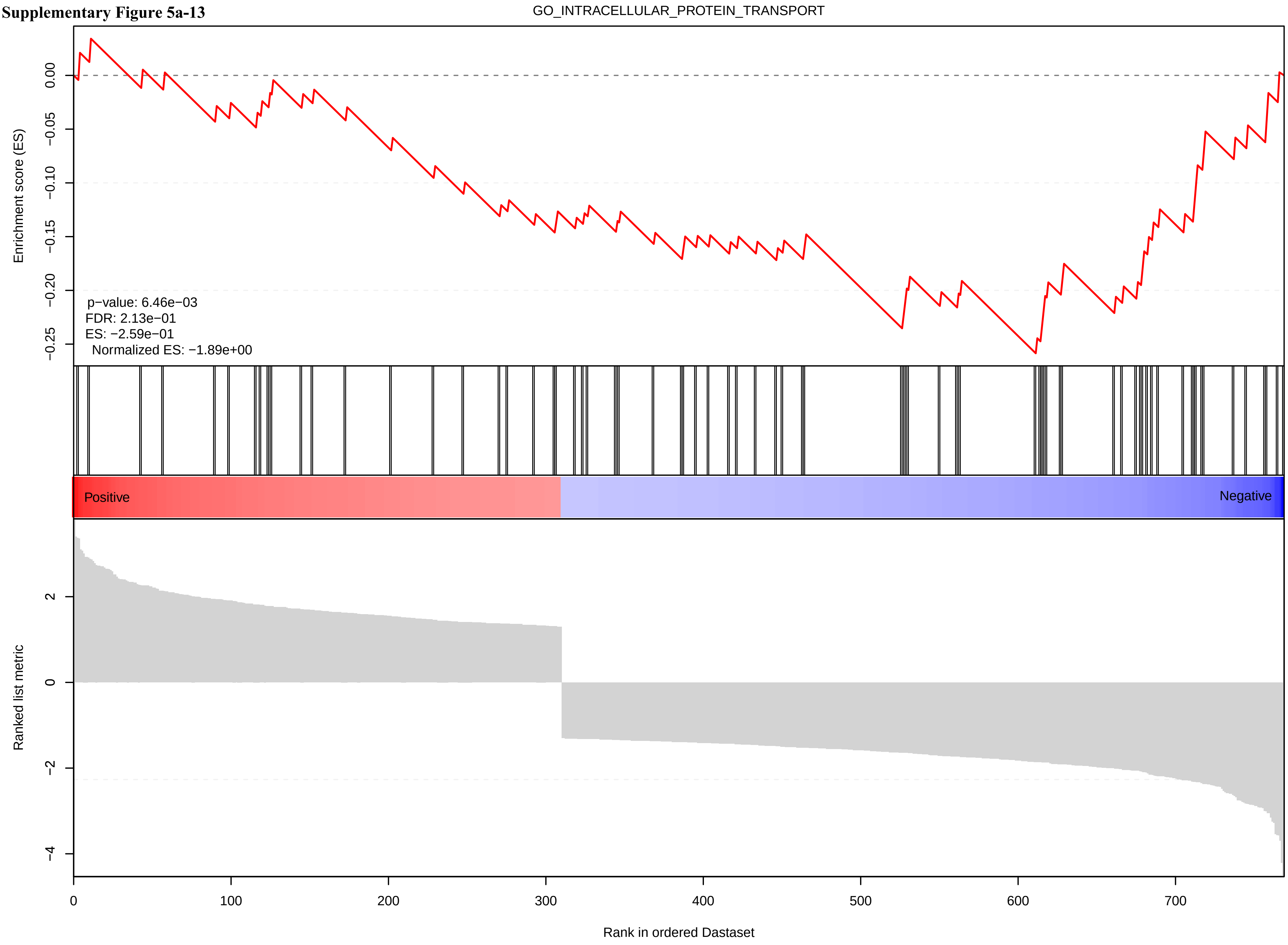

Supplementary Figure 5a-14

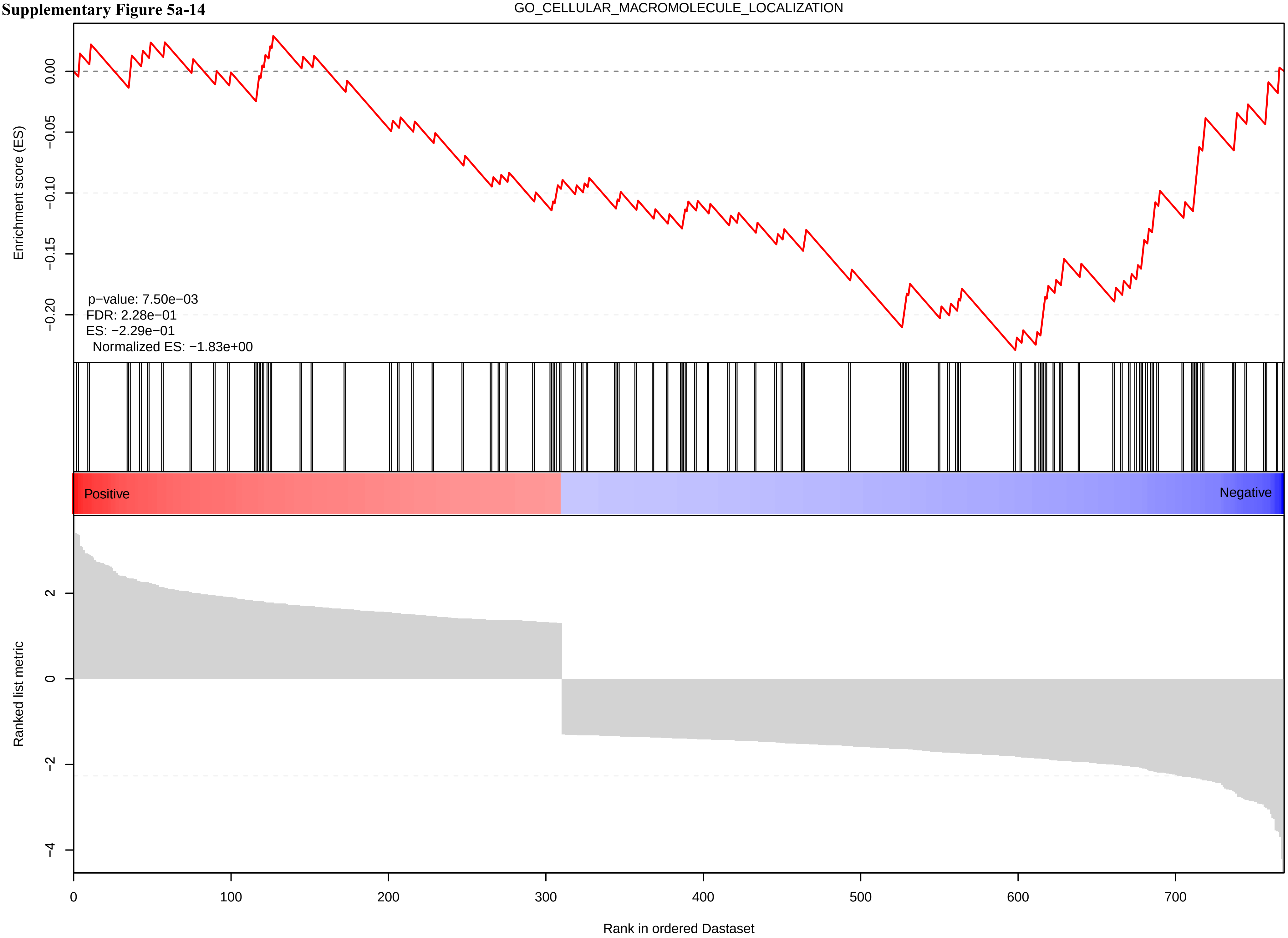

Supplementary Figure 5a-15

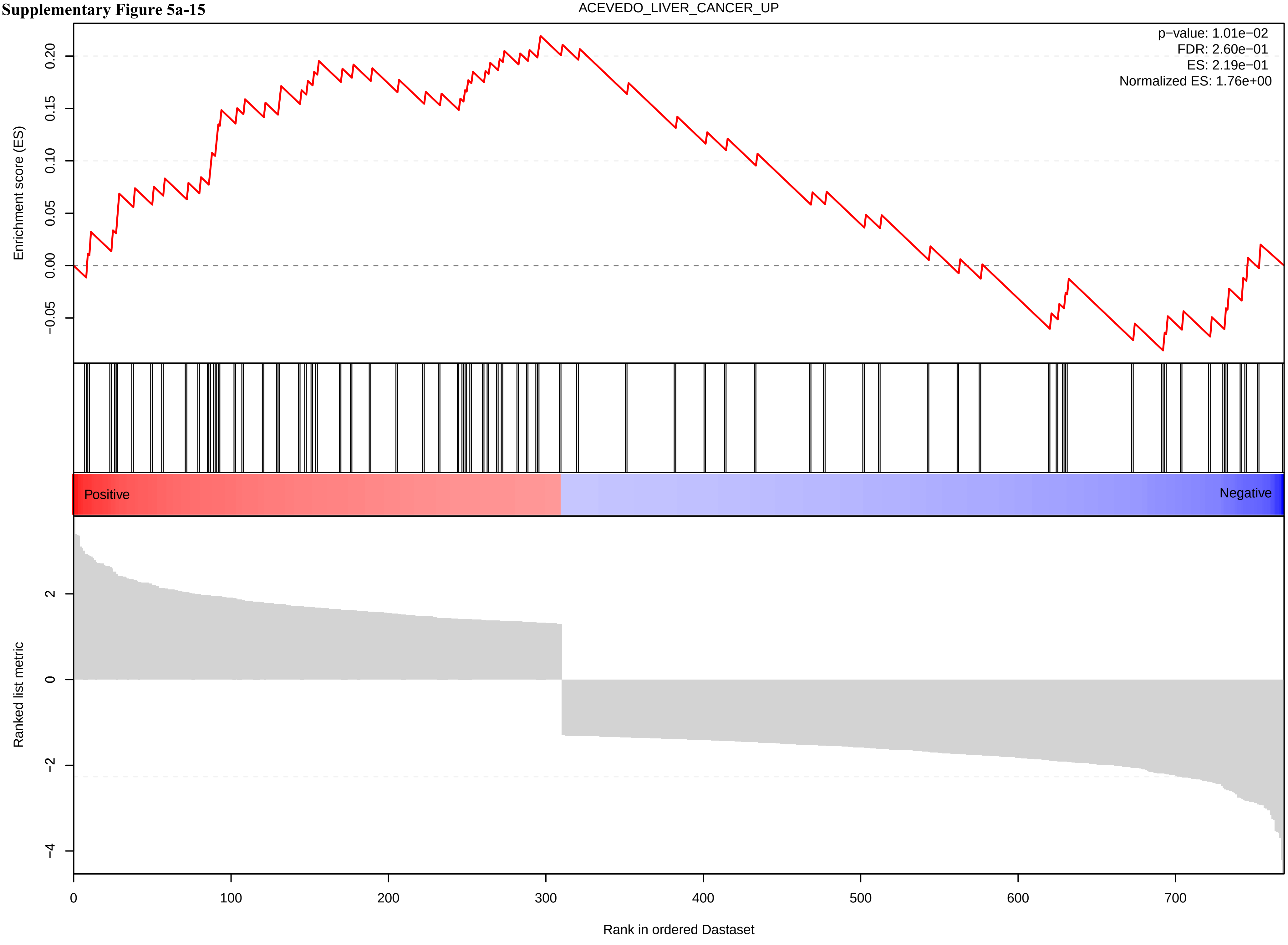

Supplementary Figure 5a-16

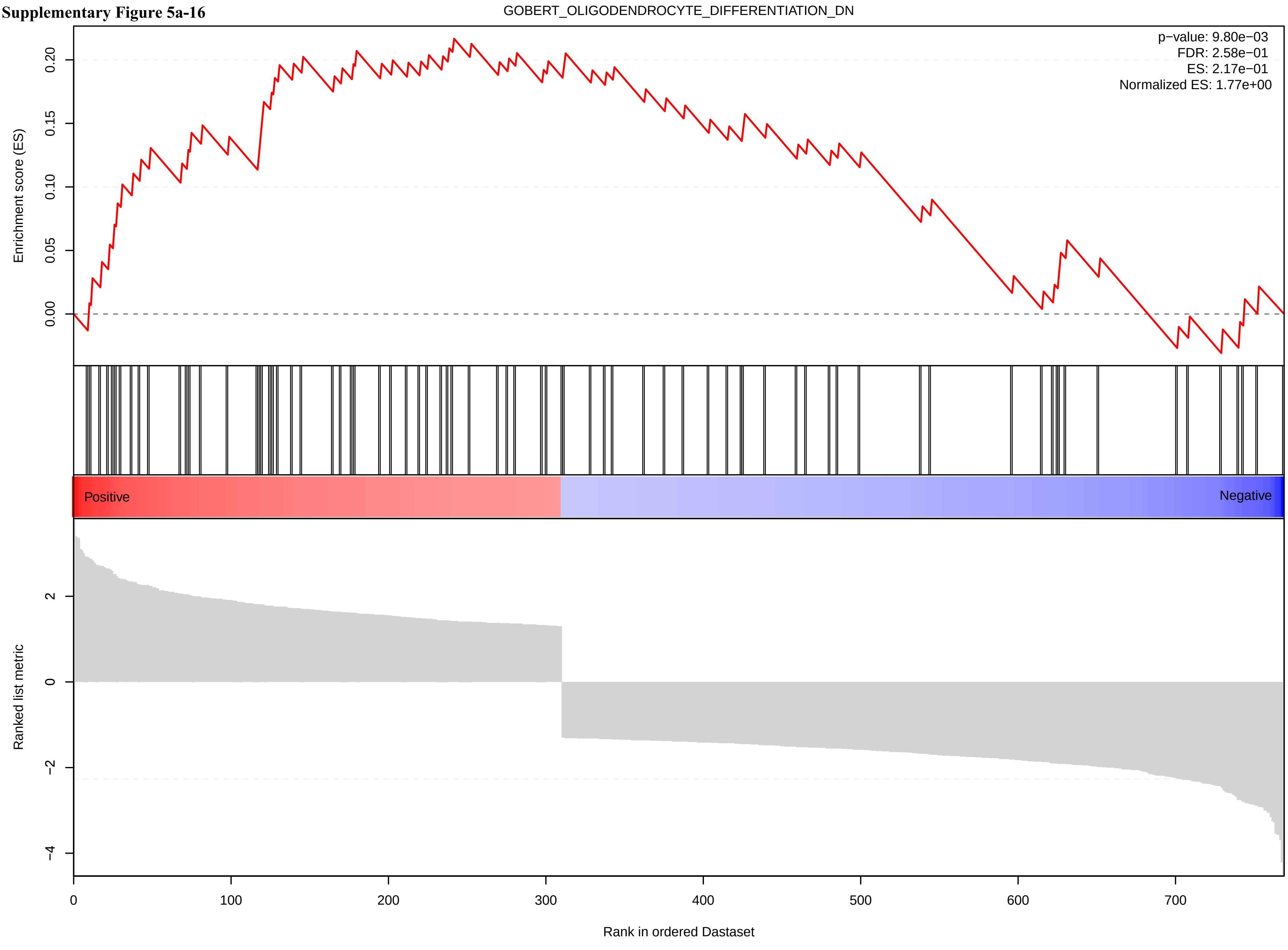

Supplementary Figure 5a-17

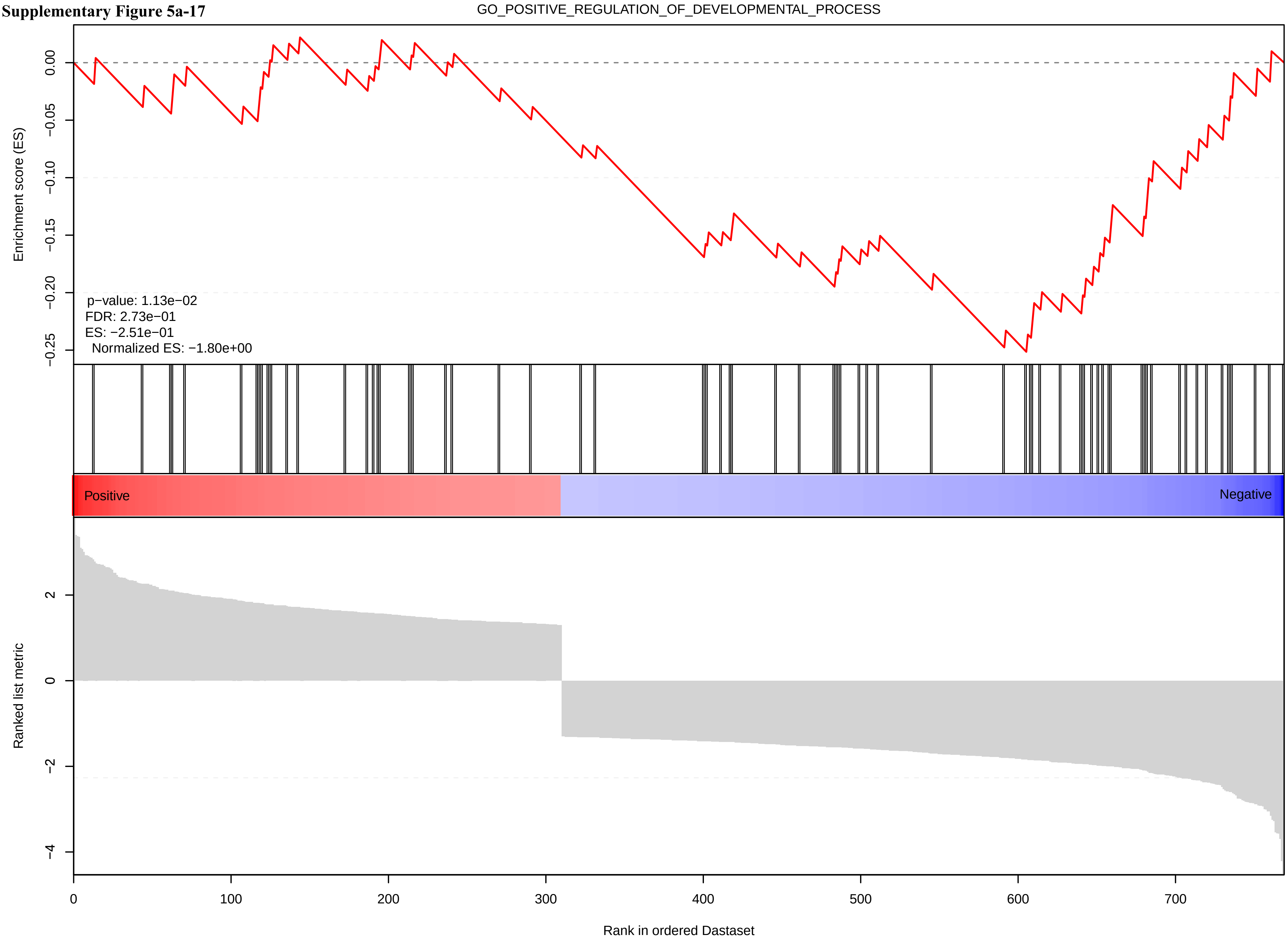

Supplementary Figure 5a-18

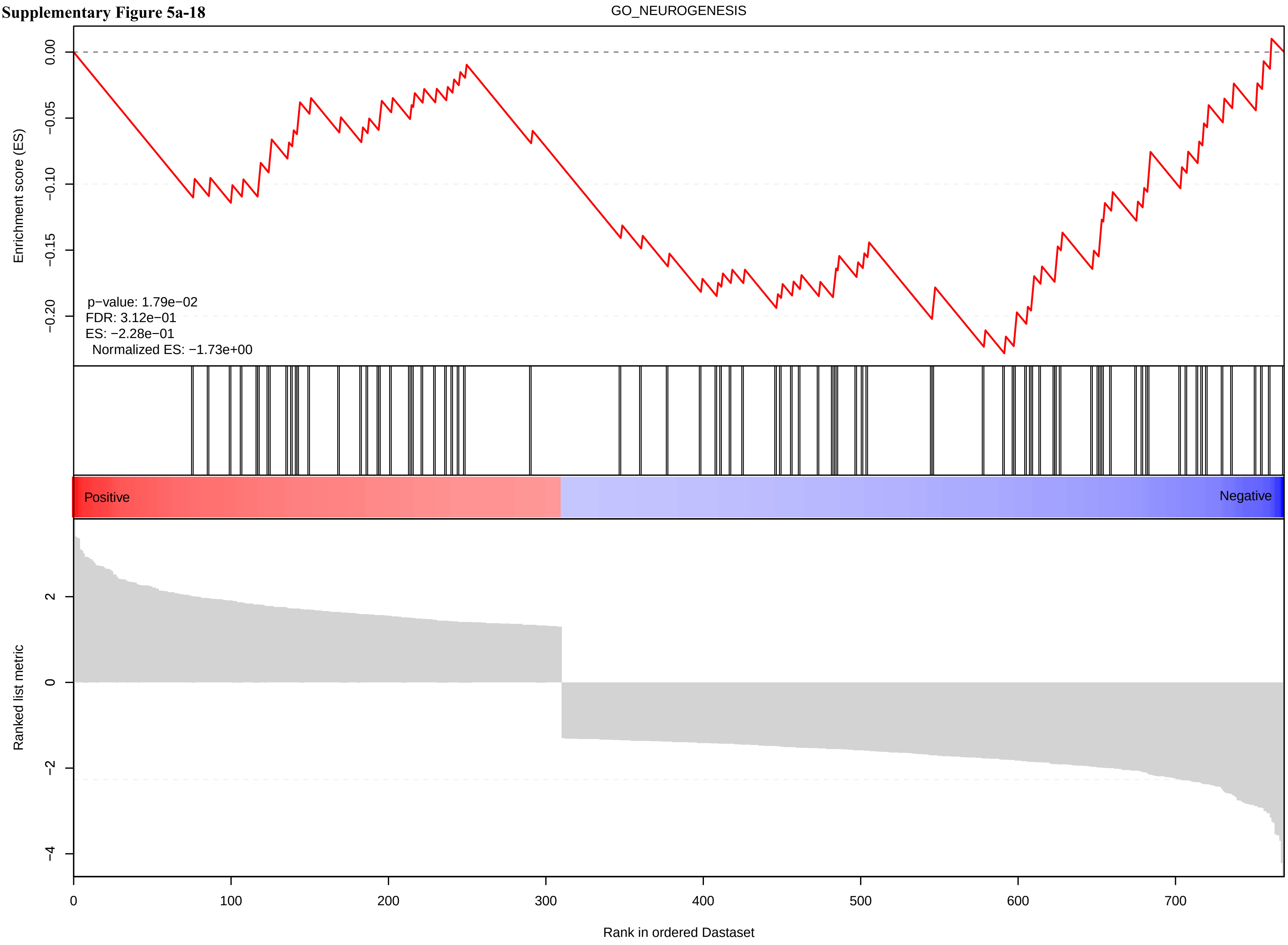

Supplementary Figure 5a-19

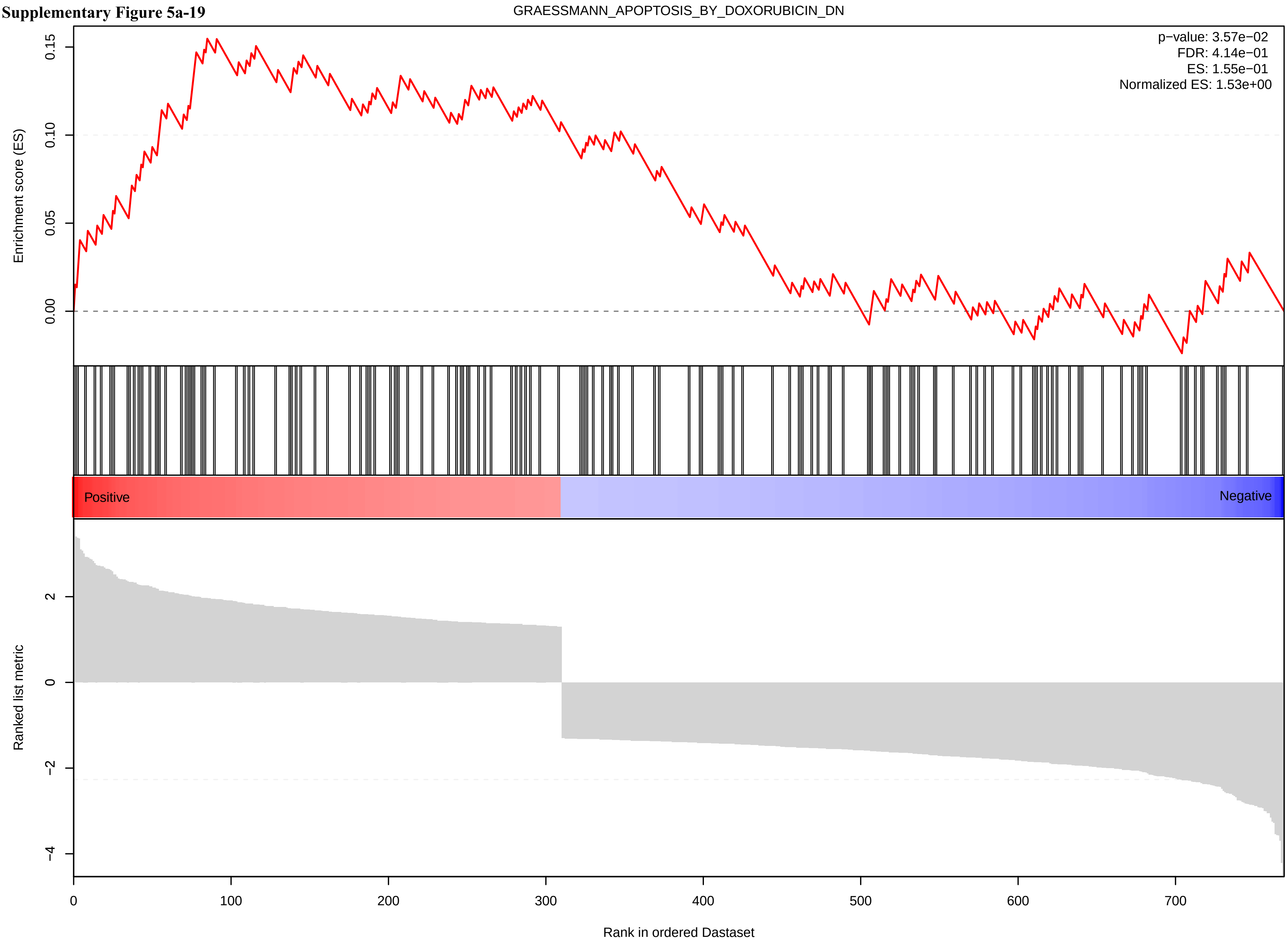

Supplementary Figure 5a-20

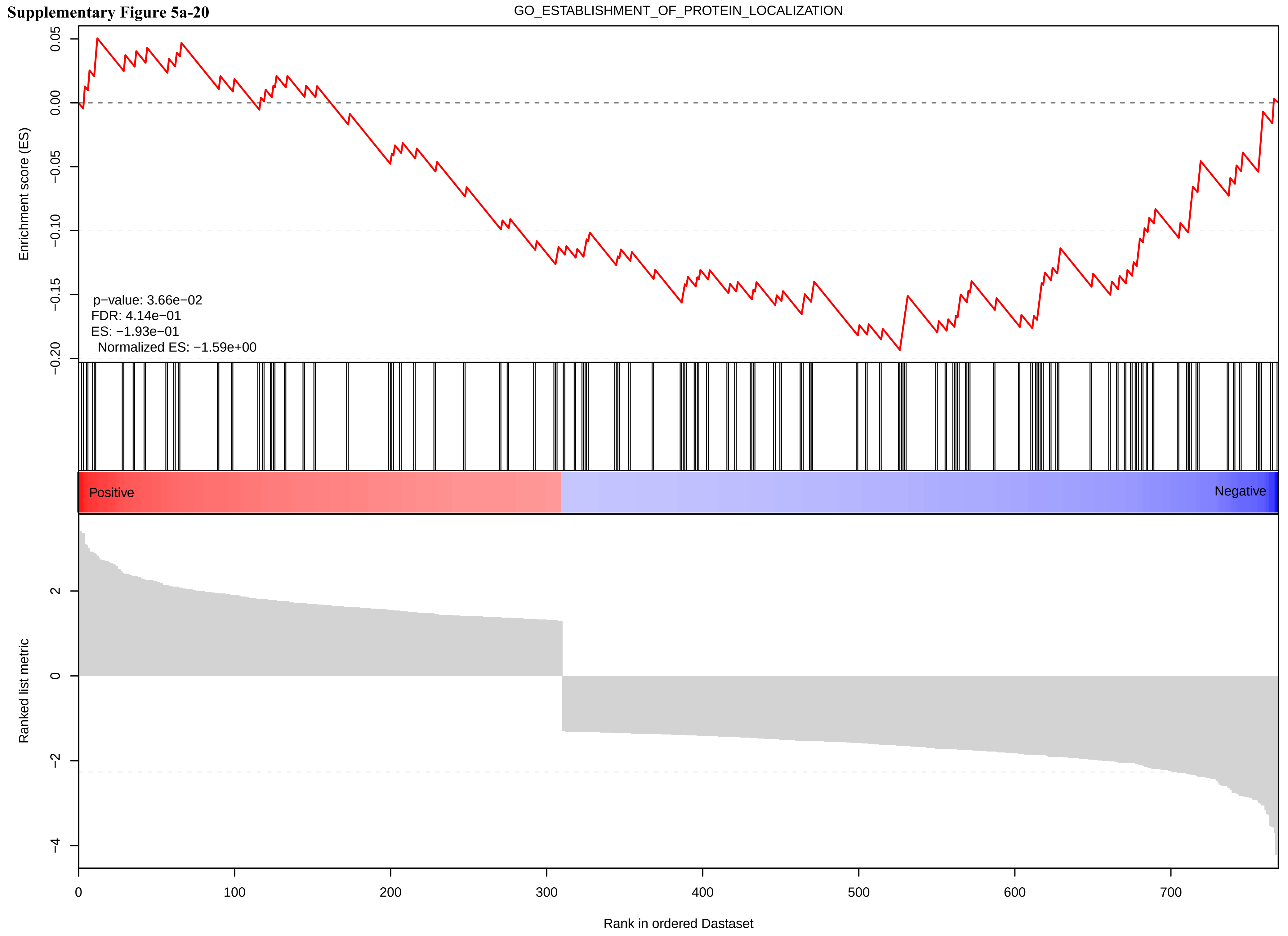

Supplementary Figure 5b-1

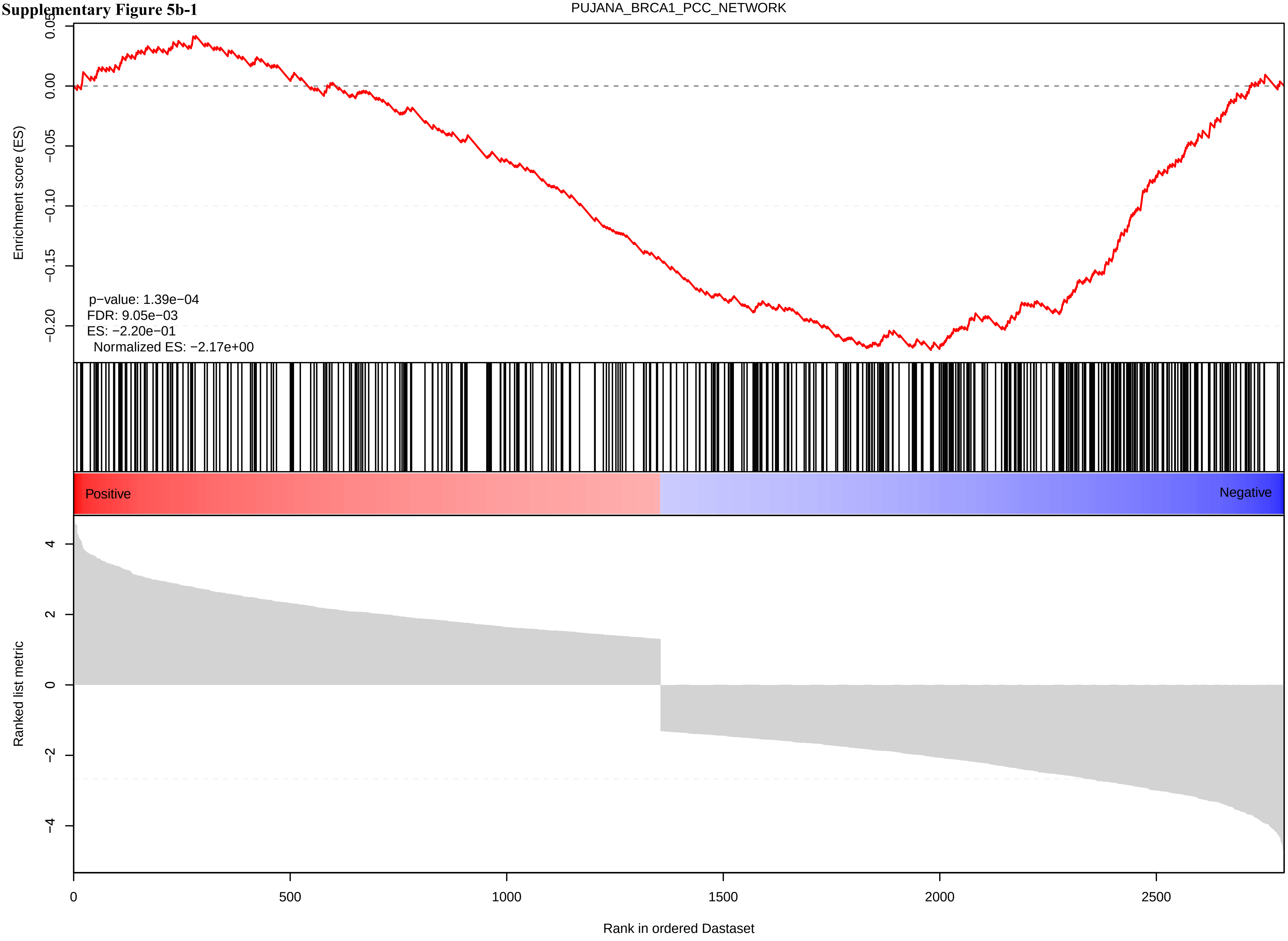

Supplementary Figure 5b-2

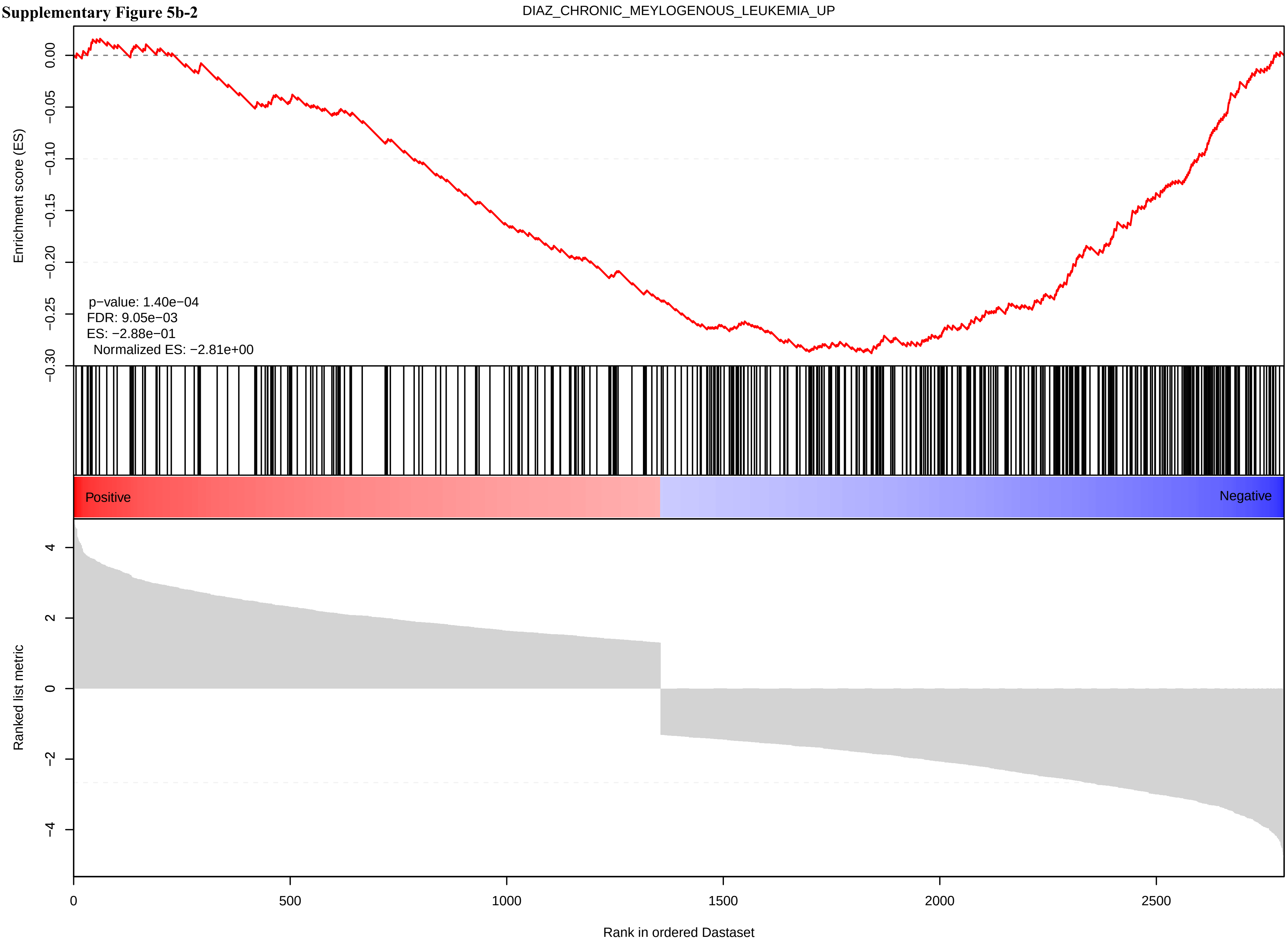

Supplementary Figure 5b-3

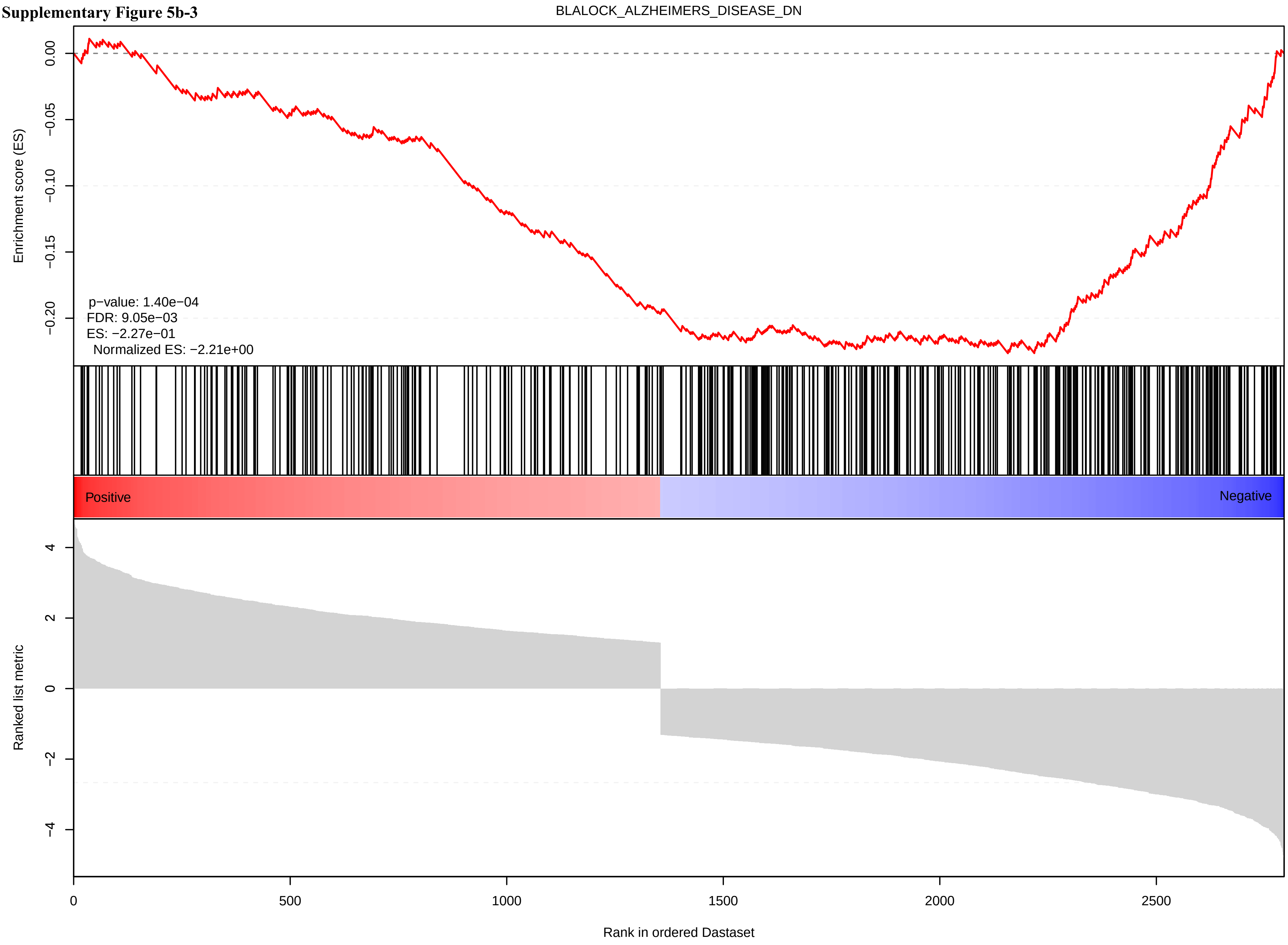

Supplementary Figure 5b-4

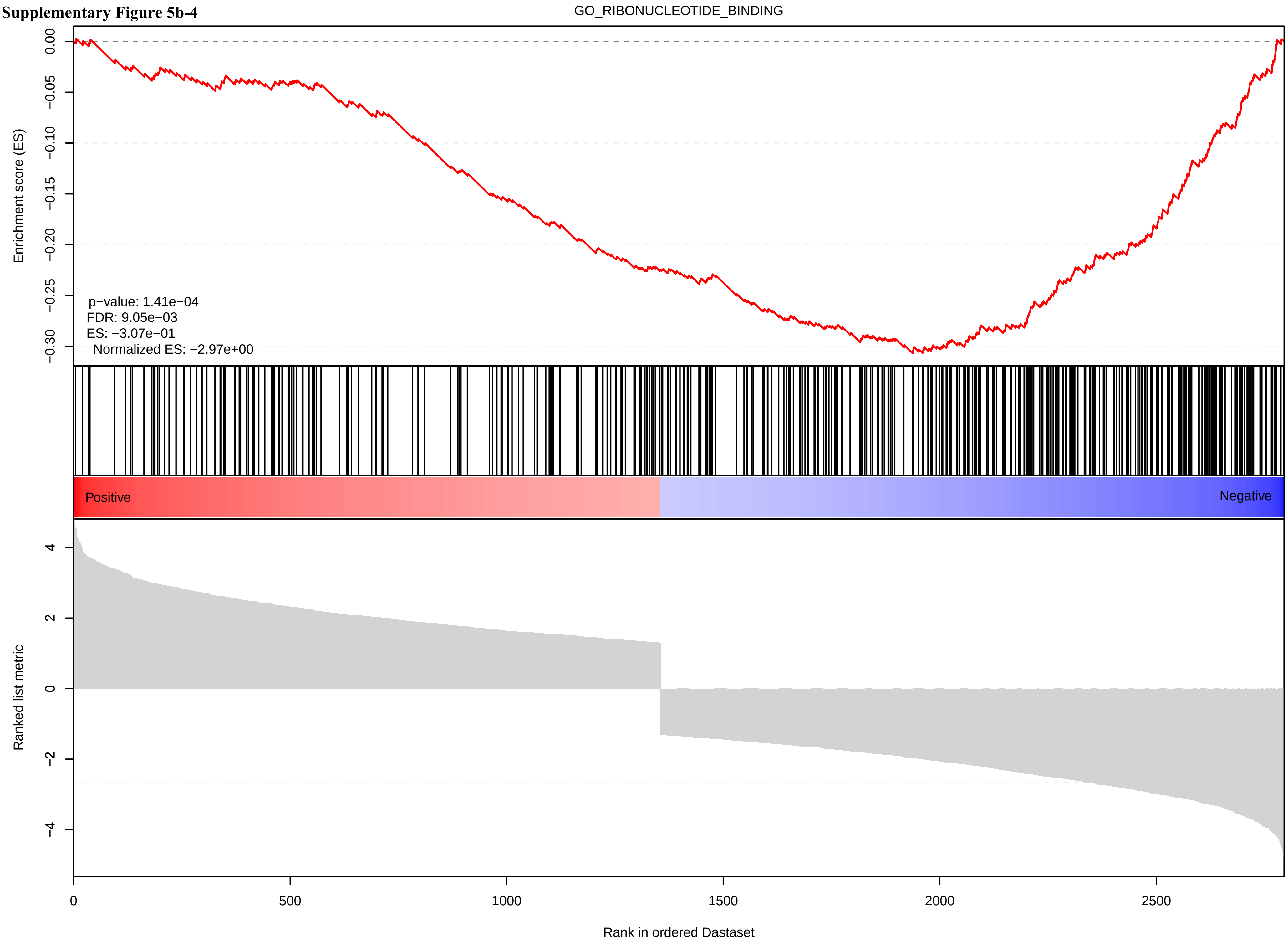

Supplementary Figure 5b-5

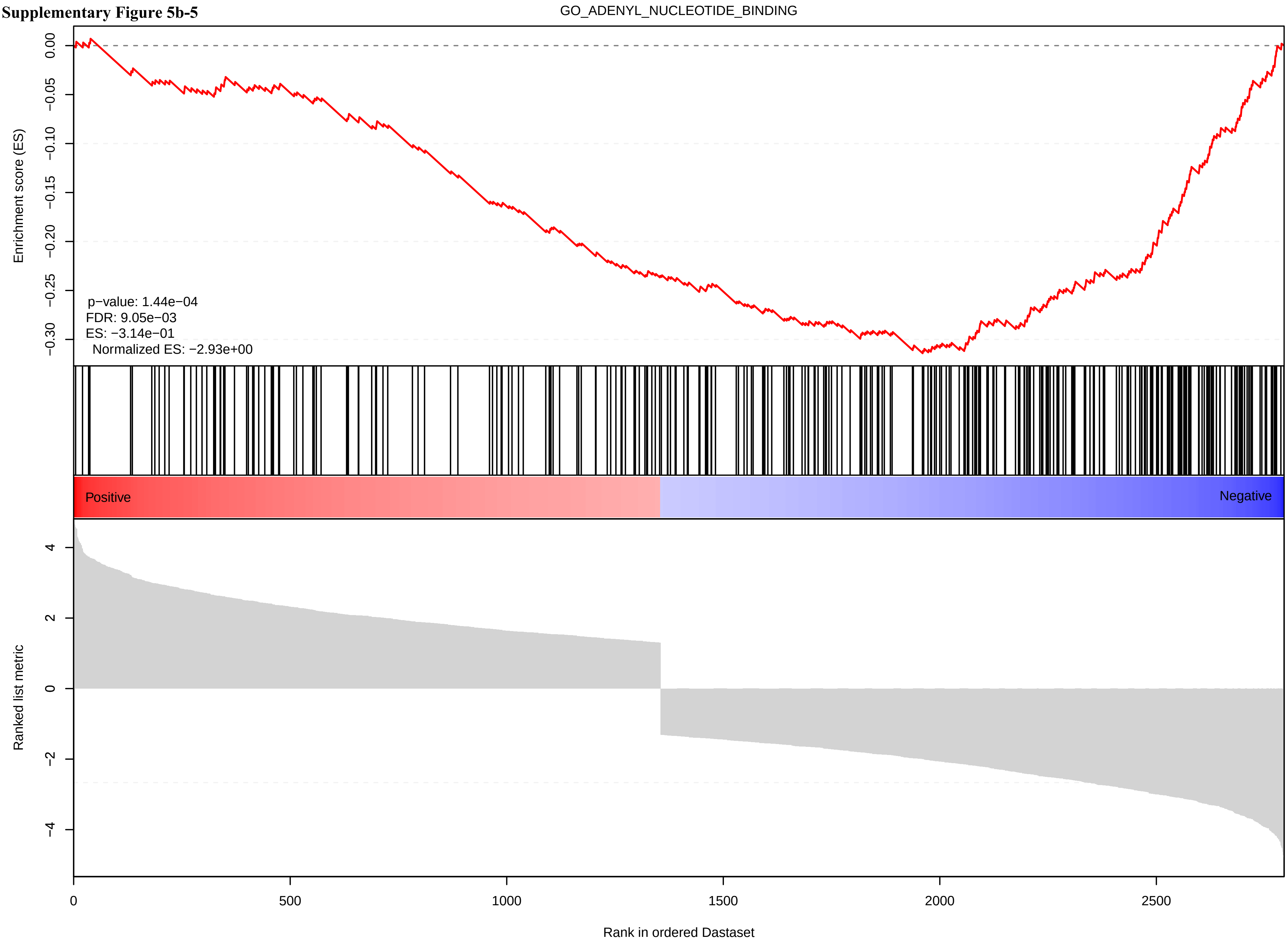

Supplementary Figure 5b-6

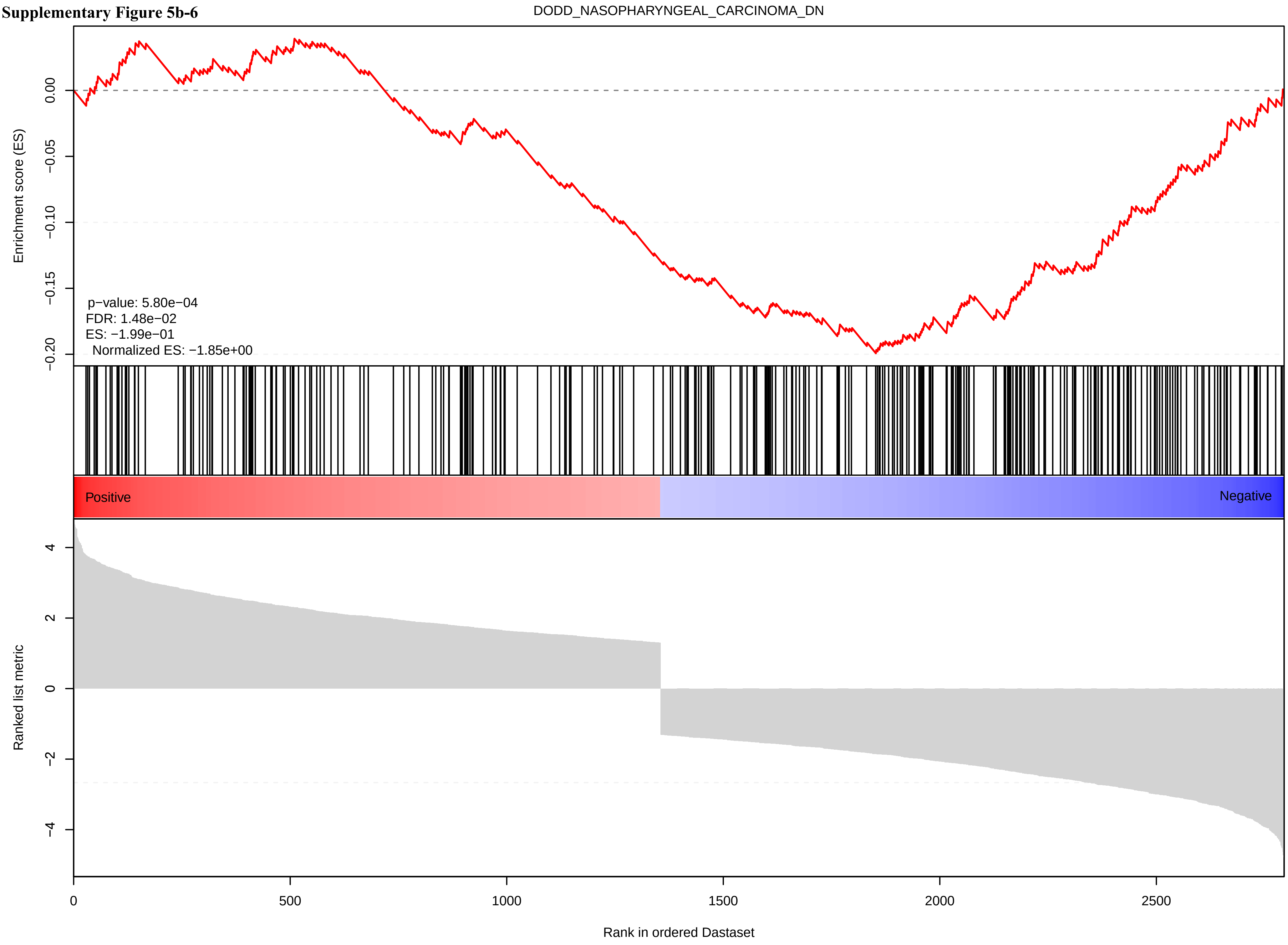

Supplementary Figure 5b-7

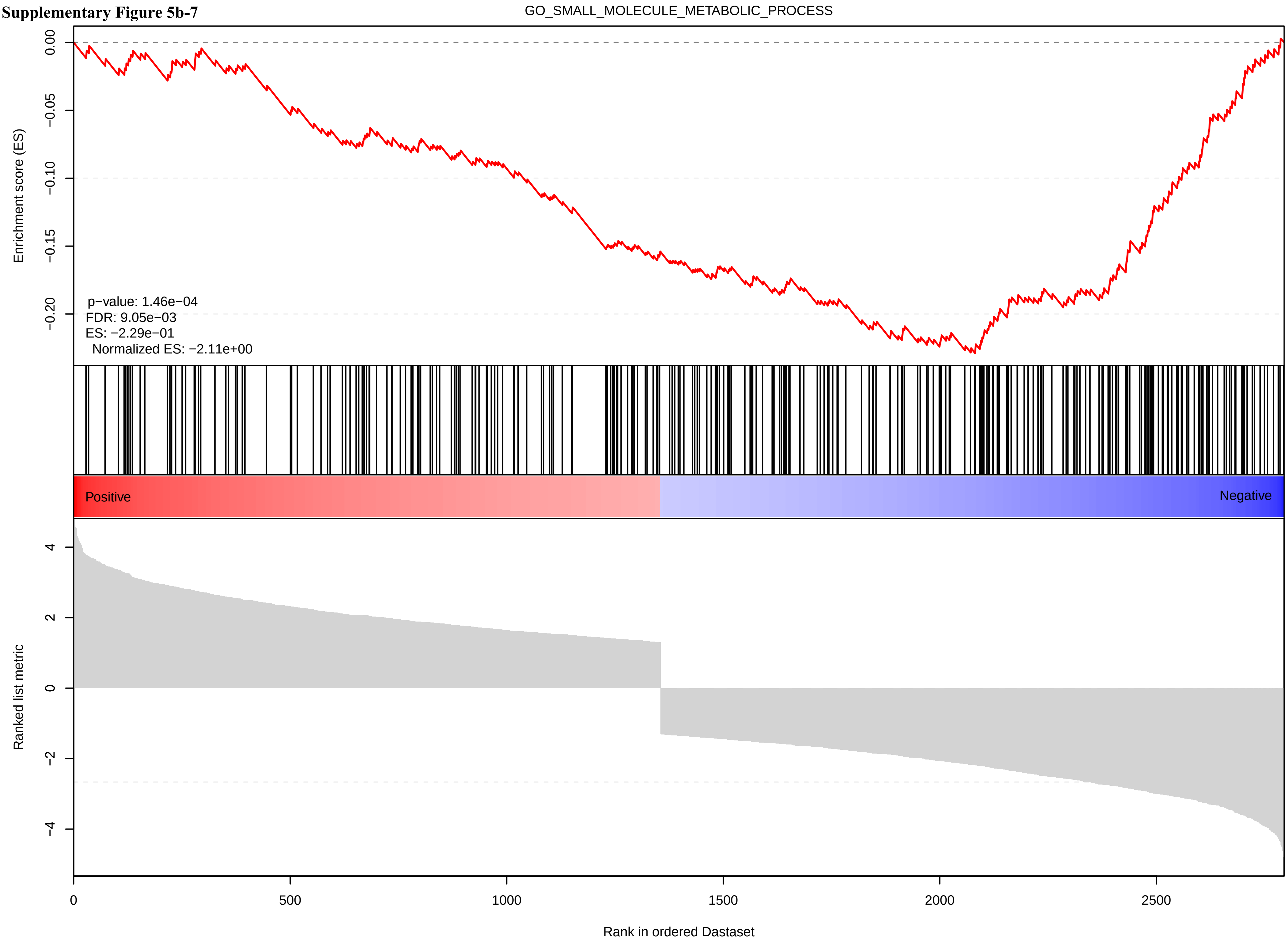

Supplementary Figure 5b-8

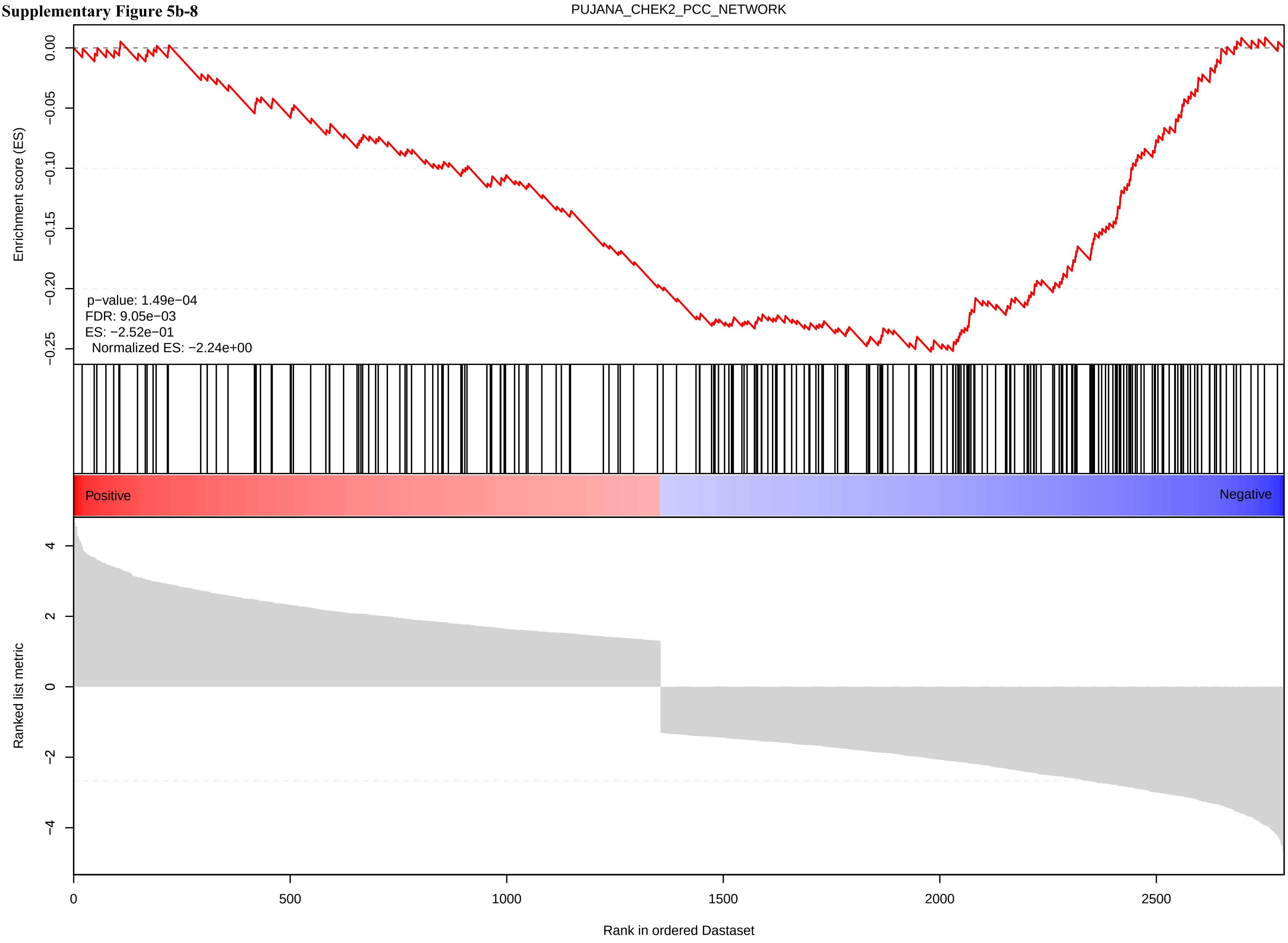

Supplementary Figure 5b-9

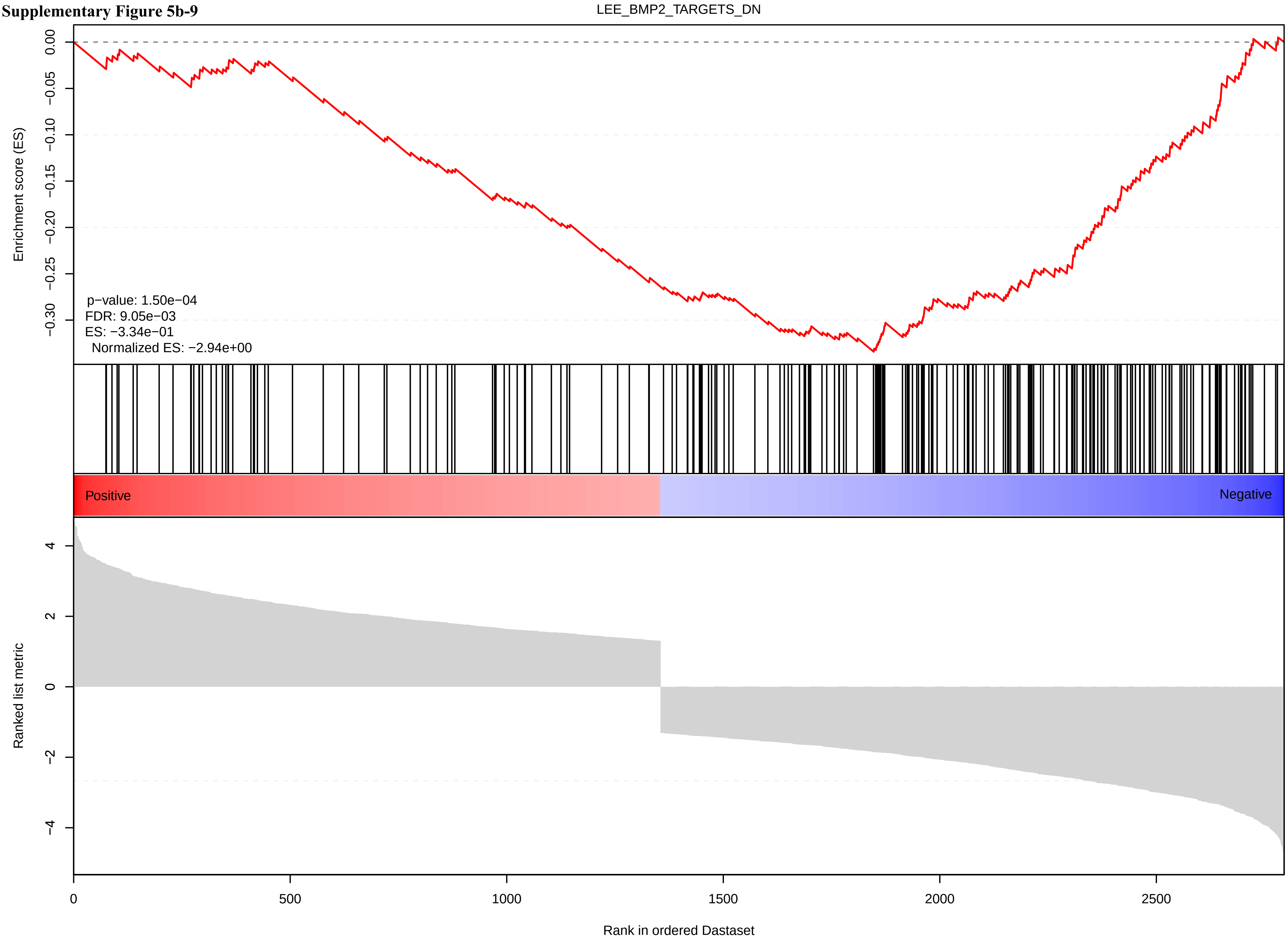

Supplementary Figure 5b-10

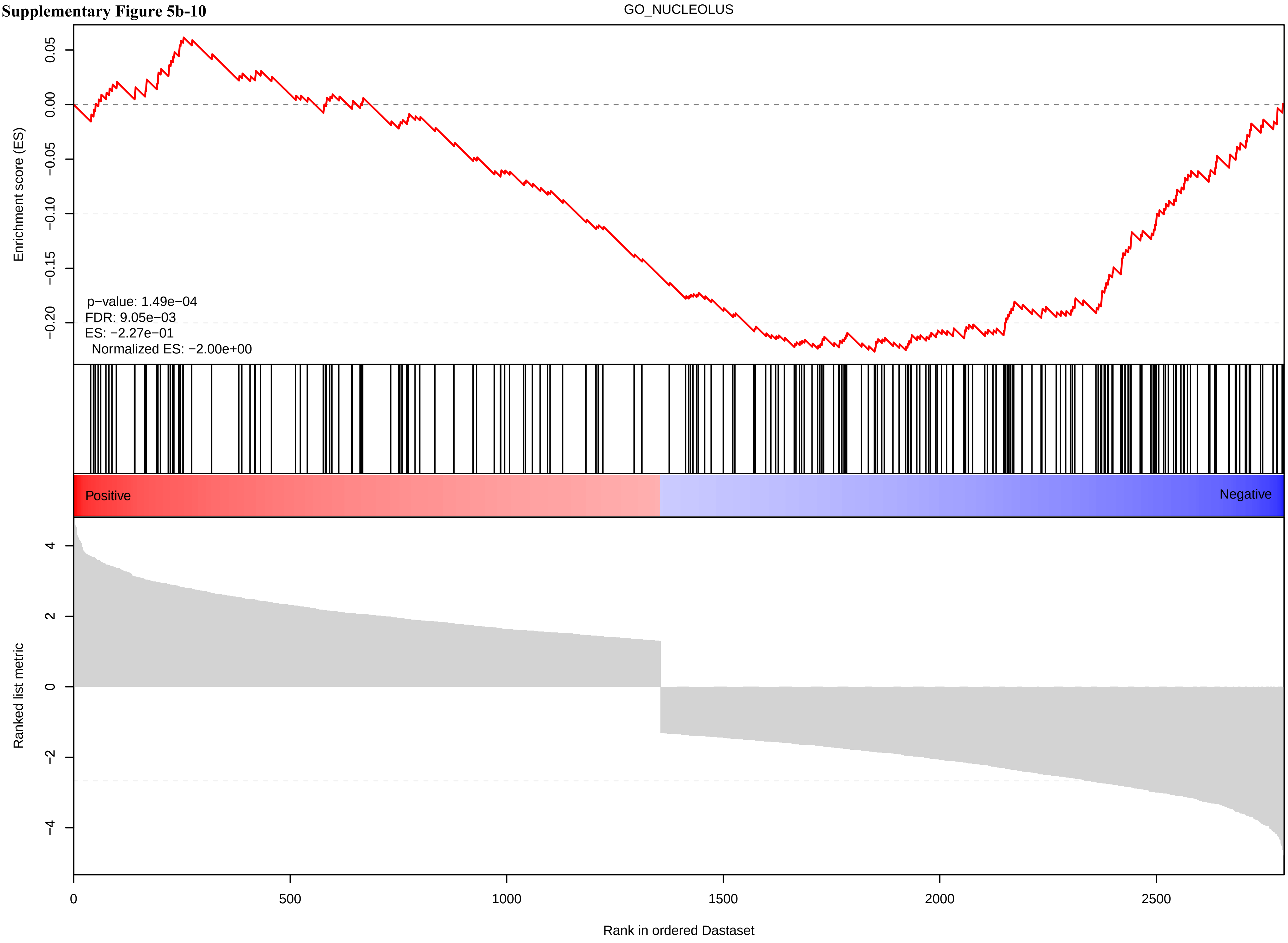

Supplementary Figure 5b-11

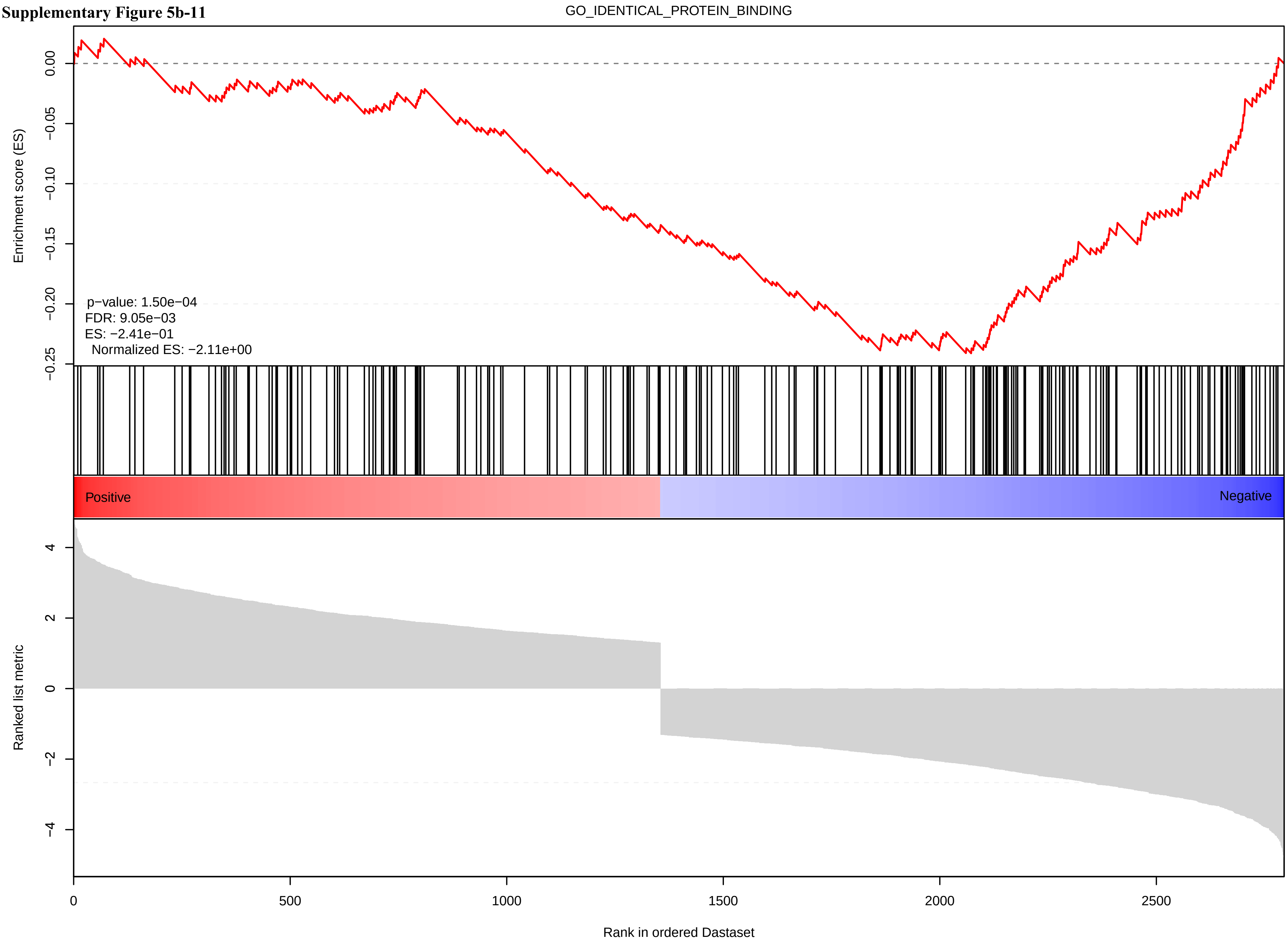

Supplementary Figure 5b-12

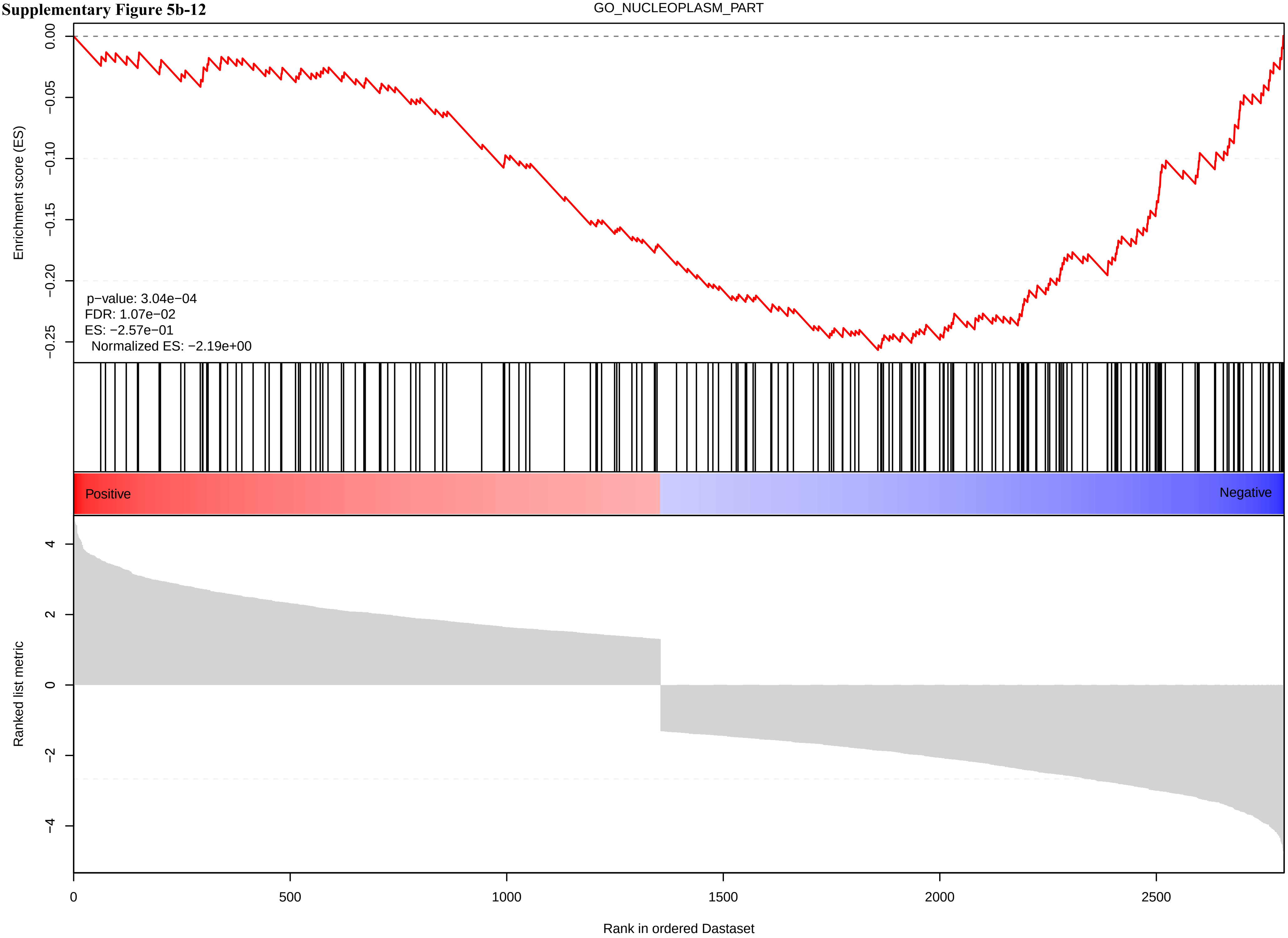

Supplementary Figure 5b-13

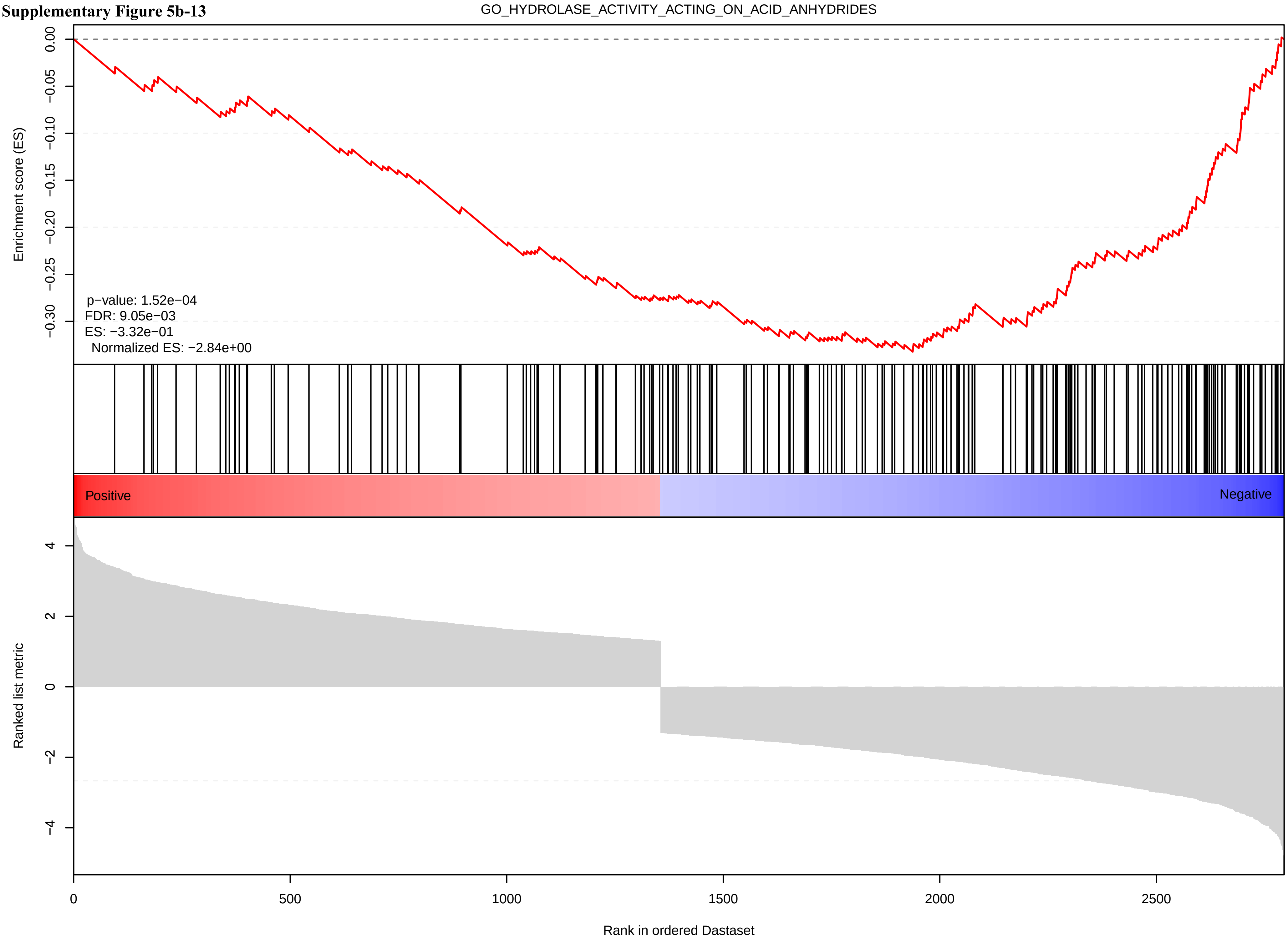

Supplementary Figure 5b-14

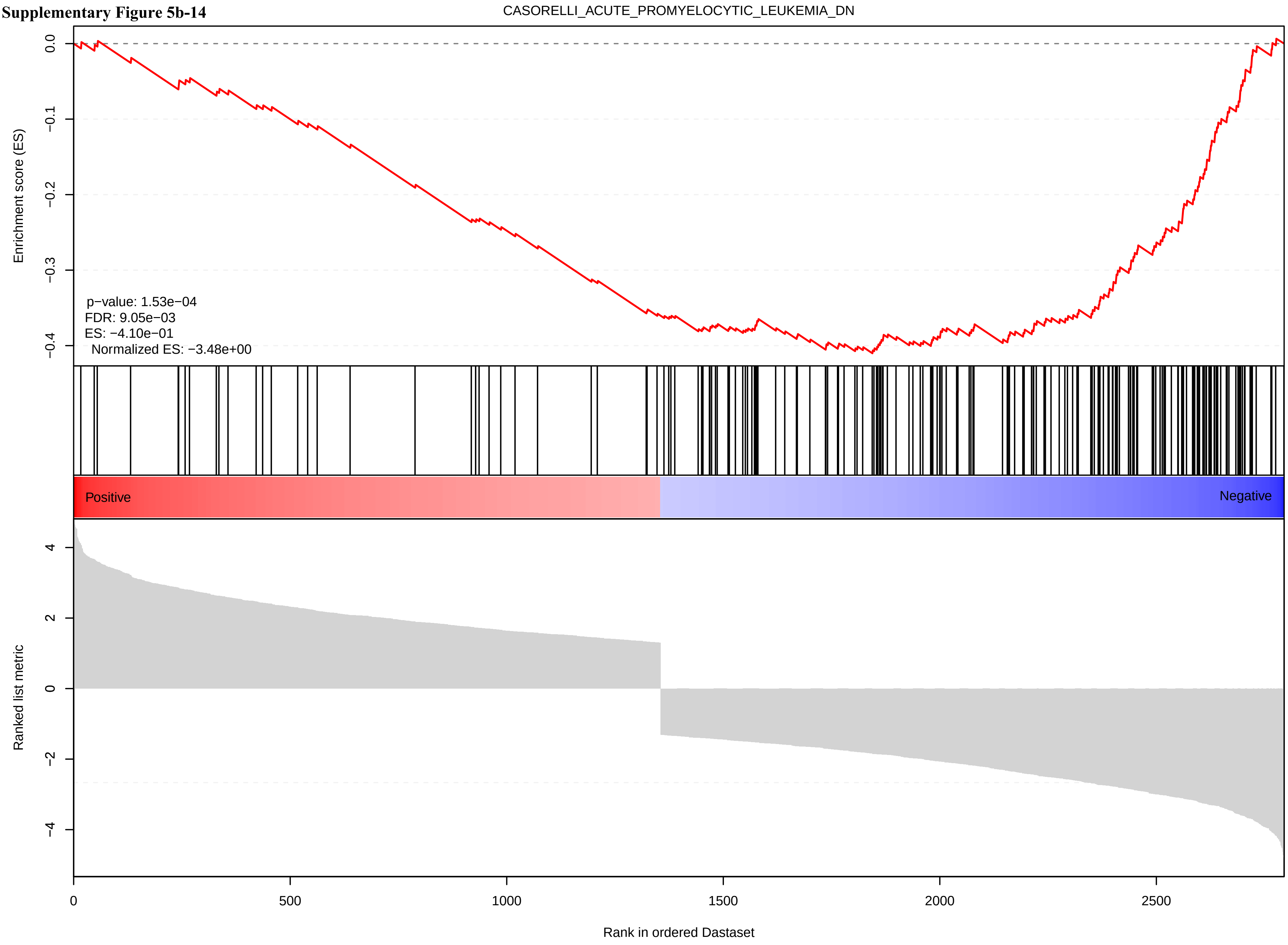

Supplementary Figure 5b-15

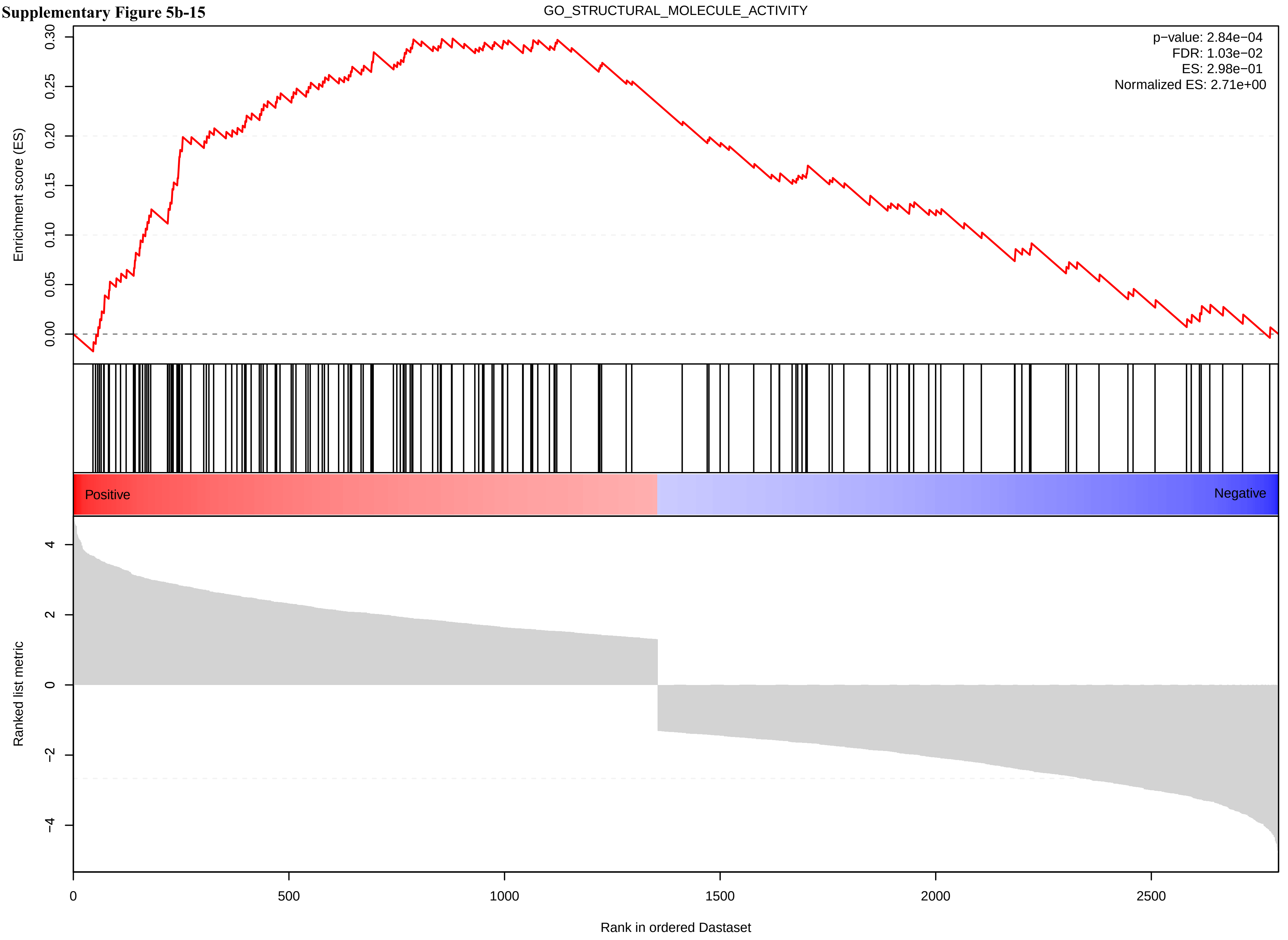

Supplementary Figure 5b-16

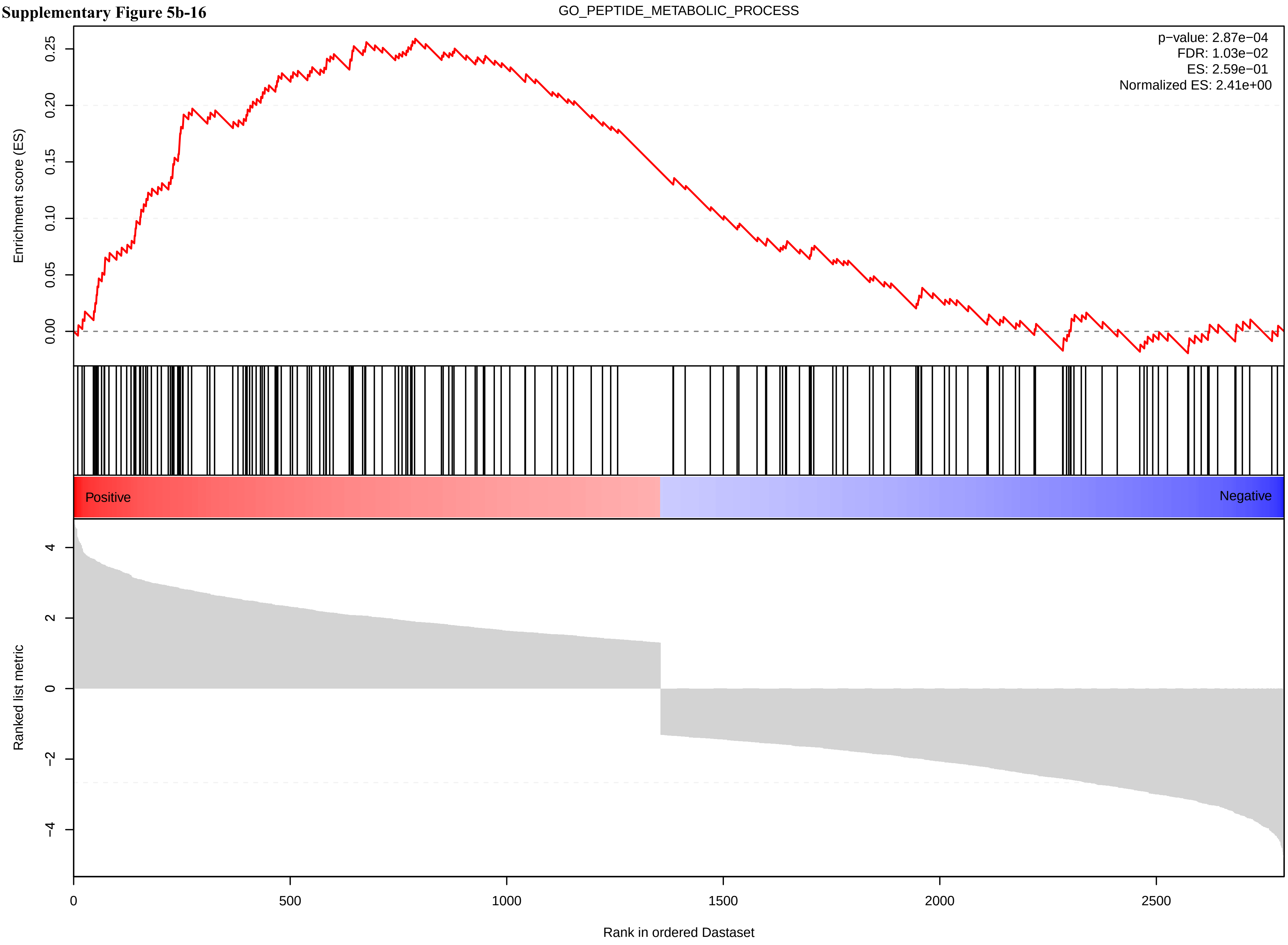

Supplementary Figure 5b-17

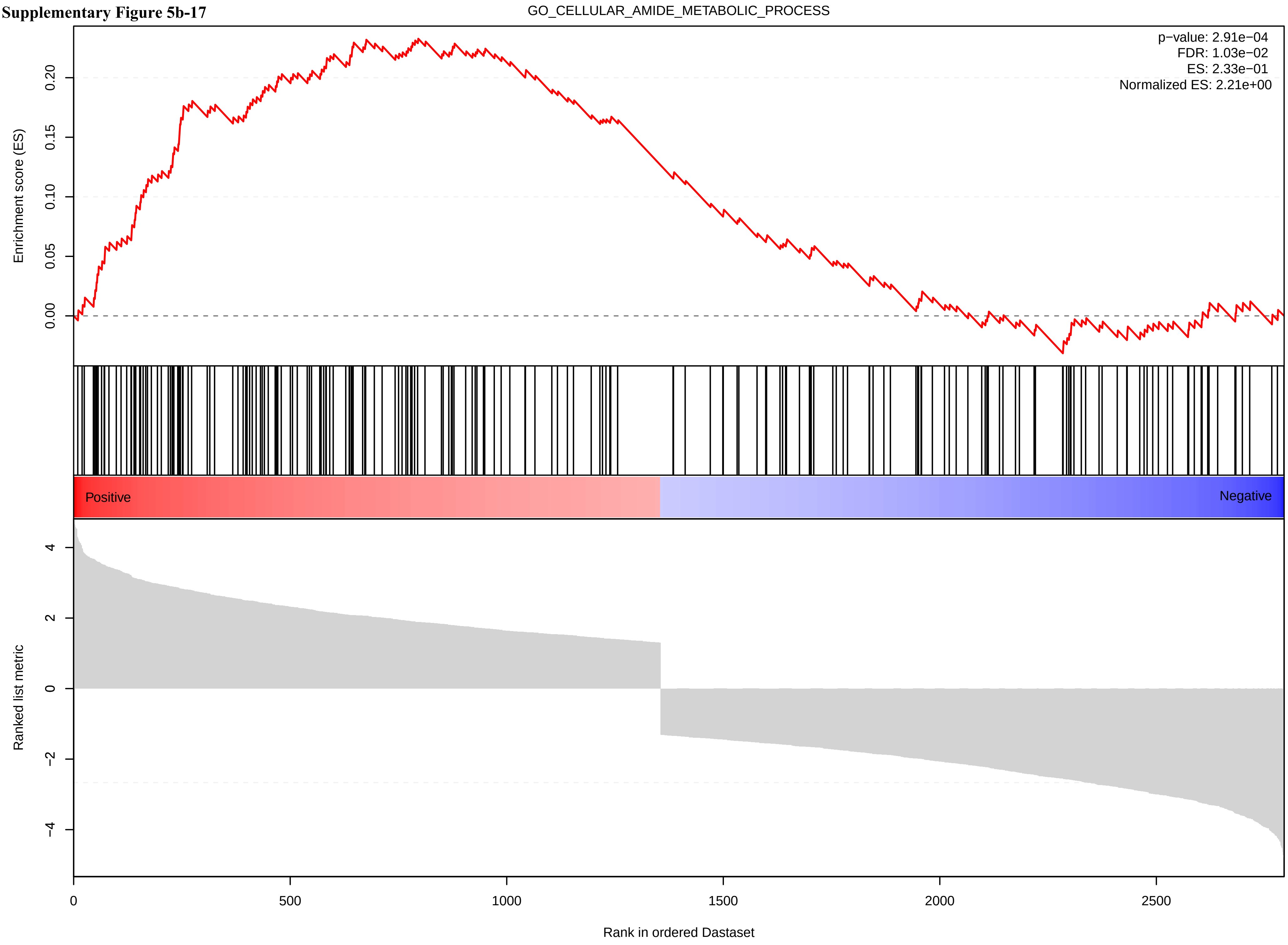

Supplementary Figure 5b-18

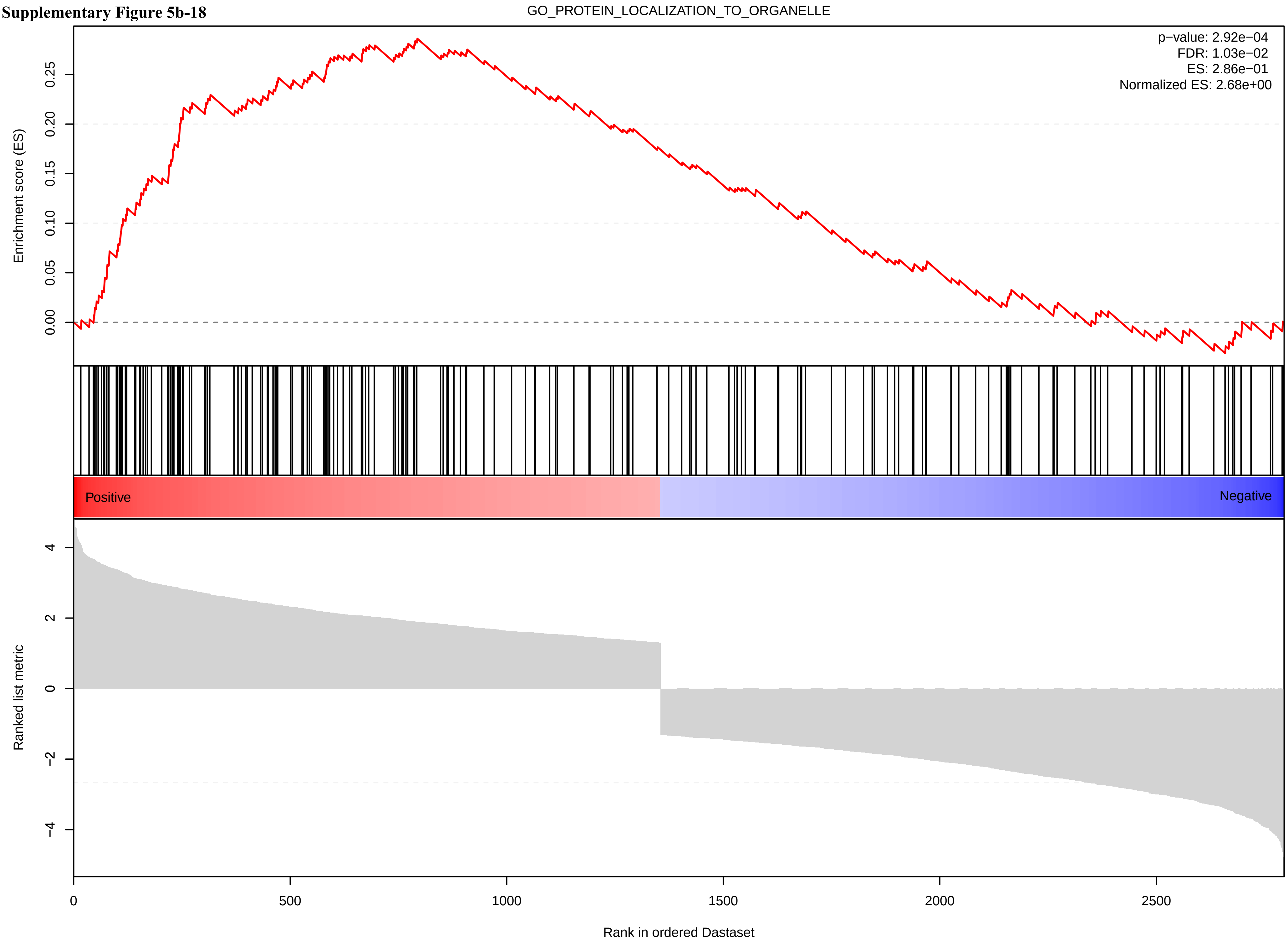

Supplementary Figure 5b-19

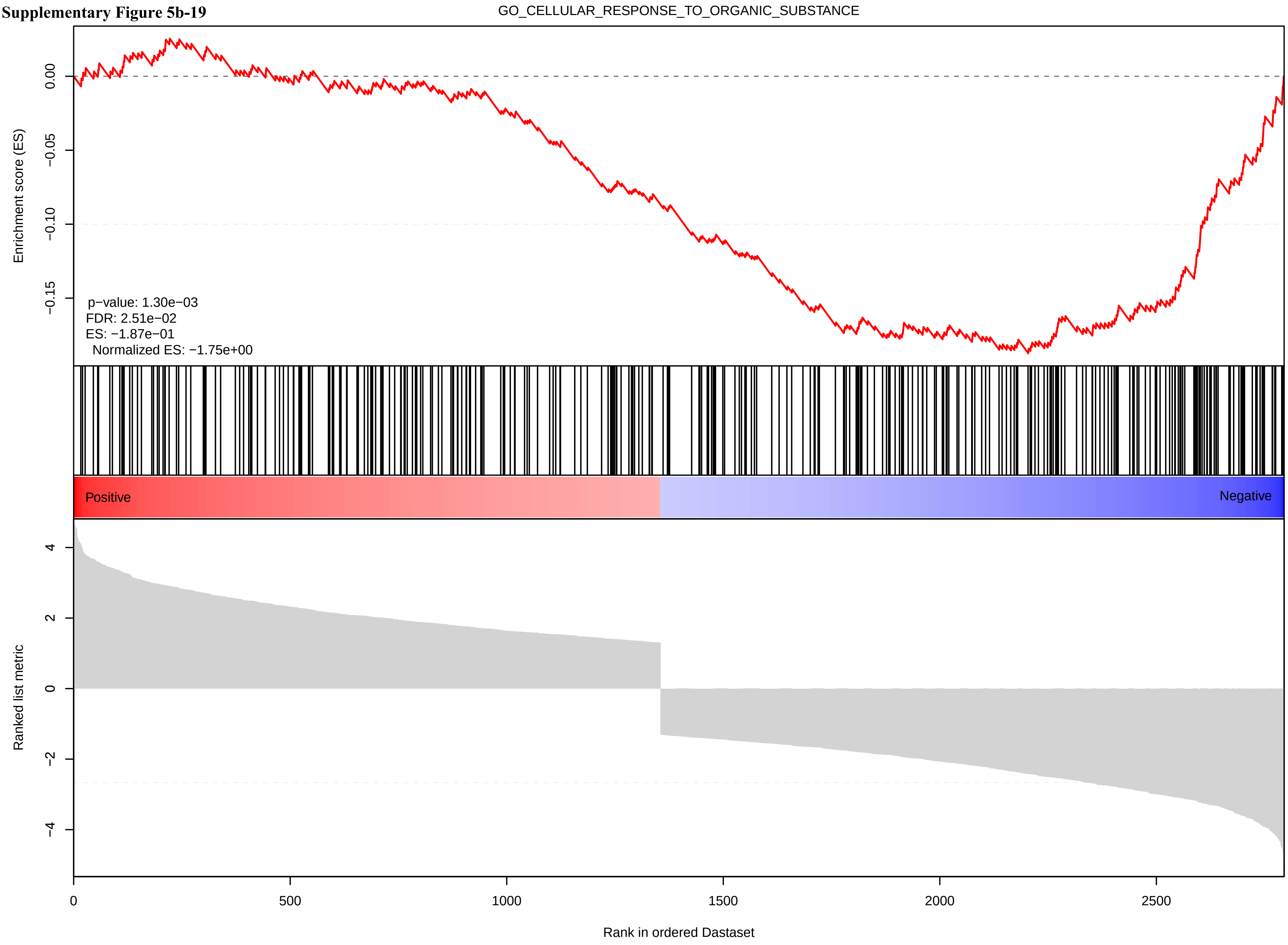

Supplementary Figure 5b-20

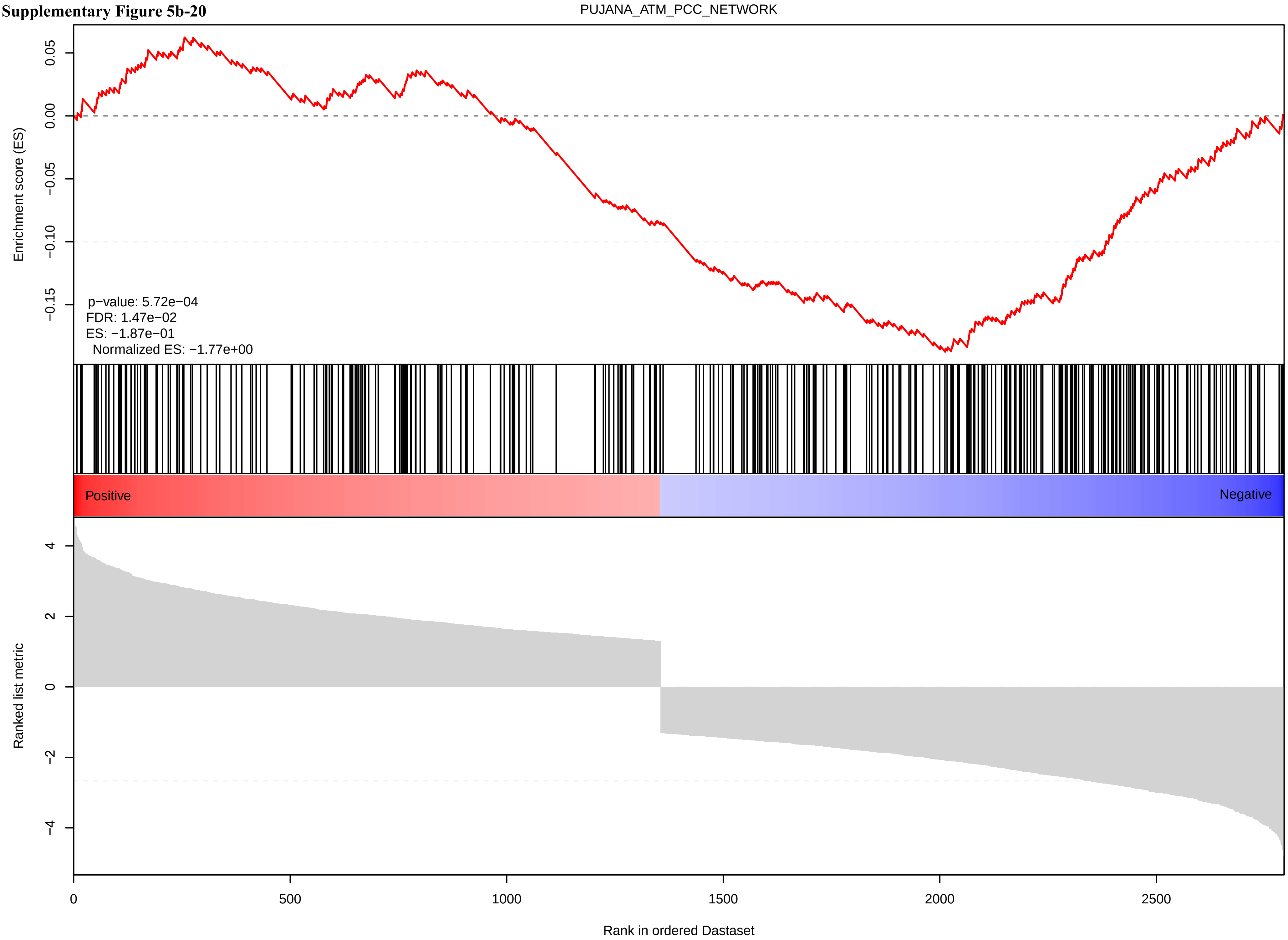

Supplementary Figure 5. Gene set enrichment analysis (GSEA) of FC- and EXT- associated differential expression across sexes. GSEA was used to test concordance of differential gene expression analyses results with gene expression signatures from the Molecular Signatures Database. Enrichment scores are calculated against background and they are also corrected for multiple comparisons Benjamini Hochberg's False Discovery Rate (FDR). Top-20 gene set enrichment analysis plots for gene sets uniquely associated with FC **(a1-20)** and EXT **(b1-20)**. The y-axis represents enrichment score (ES) and the x-axis (bar-code plot) indicates the position of the genes on the expression data rank-sorted (boxplot) by its association with FC (i.e., FC vs. TA) and EXT (i.e., EXT vs. TA), with red and blue colors indicating up- and down-regulation of RNA. ES is the maximum deviation from zero as calculated for each gene going down the ranked list and represents the degree of over-representation of a gene set at the top or the bottom of the ranked gene list.
