# Supplementary material for: Genome-wide translational profiling of amygdala Crh-expressing neurons reveals role for CREB in fear extinction learning
Source: Nat Commun. 2020 Oct 14;11:5180. doi: 10.1038/s41467-020-18985-6 (PMC7560654; doi:10.1038/s41467-020-18985-6)
Supplement: Supplementary file 3 — Description of Additional Supplementary Files [file 41467_2020_18985_MOESM3_ESM.pdf]

Description of Additional Supplementary Files

File name: Supplementary Data 1
Description: This file contains the differentially expressed genes (DEG) according to the three pairwise comparisons: Fear Conditioning (FC) vs. Tone Alone (TA), Extinction (EXT) vs. TA, and EXT vs. FC in male (A-C), female (D-F), and combined male and female (G-I). Analysis was performed using limma with False Discovery Rate (FDR) multiple testing correction of p-values.

File name: Supplementary Data 2
Description: This file contains the network analysis results based on the weighted gene co-expression network analysis (WGCNA) software. This file annotates genes (Column A) according to: i) the module they belong (Column B), ii) their connectivity to all other genes (kTotal – Column C)), iii) their connectivity to all other genes within the module they belong (kWithin – Column D), iii) the difference between kTotal and KWithin (kOut – Column E), iv) the difference between kWithin and kOut (kDiff – Column F), v) their correlation (r-value) with module eigengenes (Columns G to Z), and the respective Student's asymptotic p-value (Columns AA to AT).

File name: Supplementary Data 3
Description: This file contains the gene set enrichment analysis (GSEA) of FC associated differential expression across sexes. GSEA was used to test concordance of differential gene expression analyses' results with gene expression signatures from the Molecular Signatures Database. This file includes: i) the name of the pathway (Column A), ii) the Enrichment scores (ES), which is the maximum deviation from zero encountered in the ranked FC gene list (ES - Column B), iii) the enrichment p-values and the corresponding FDR-adjusted p-value (Columns C and D, respectively), iv) the normalized ES (NES), which is the actual ES divided by the mean enrichment for random gene sets with the same size as the target gene set (Column E), v) the number of times a random gene set had a more extreme ES (nMoreExtreme) (Column F), and vi) the size of the target gene set (Column G).

File name: Supplementary Data 4
Description: This file contains the gene set enrichment analysis (GSEA) of EXT associated differential expression across sexes. GSEA was used to test concordance of differential gene expression analyses' results with gene expression signatures from the Molecular Signatures Database. This file includes: i) the name of the pathway (Column A), ii) the Enrichment scores (ES), which is the maximum deviation from zero encountered in the ranked EXT gene list (ES-Column B), iii) the enrichment p-values and the corresponding FDR-adjusted p-value (Columns-C and D, respectively), iv) the normalized ES (NES), which is the actual ES divided by the mean enrichment for random gene sets with the same size as the target gene set (Column E), v) the number of times a random gene set had a more extreme ES (nMoreExtreme) (Column F), and vi) the size of the target gene set (Column G).

File name: Supplementary Data 5
Description: This file contains the Upstream regulator analysis (URA) of genes uniquely associated with FC or EXT or included in modules uniquely associated with FC or EX. URA

was performed at default setting to estimate a set of significant (Fisher's exact test p-value<0.05) deactivated (z-score<-2) or activated (z-score>2) upstream regulators. This file includes: i) name of the input data set (Column A), ii) the upstream regulator name (Column B), iii) the molecular type of the upstream regulator (Column C), iv) the predicted activation state (Column D), v) the predicted z-score (Column E), the bias-corrected z-score (Column F) and their respective average (Column G), vi) enrichment p-value and the respective FDR corrected p-value (Columns H and I, respectively), and vii) the molecules in input dataset targeted by the upstream regulator (Column J).
