# Supplementary material for: Genome-wide translational profiling of amygdala Crh-expressing neurons reveals role for CREB in fear extinction learning
Source: Nat Commun. 2020 Oct 14;11:5180. doi: 10.1038/s41467-020-18985-6 (PMC7560654; doi:10.1038/s41467-020-18985-6)
Supplement: Supplementary file 9 — Reporting Summary [file 41467_2020_18985_MOESM9_ESM.pdf]

nature research

Corresponding author(s): Ressler, Daskalakis

updated by author(s): 2020-09-19

Reporting Summary

Nature Research wishes to improve the reproducibility of the work that we publish. This form provides structure for consistency and transparency in reporting. For further information on Nature Research policies, see our Editorial Policies and the Editorial Policy Checklist.

Statistics

For all statistical analyses, confirm that the following items are present in the figure legend, table legend, main text, or Methods section.

n/a	Confirmed	
☐	☒	The exact sample size (*n*) for each experimental group/condition, given as a discrete number and unit of measurement
☐	☒	A statement on whether measurements were taken from distinct samples or whether the same sample was measured repeatedly
☐	☒	The statistical test(s) used AND whether they are one- or two-sided<br>*Only common tests should be described solely by name; describe more complex techniques in the Methods section.*
☐	☒	A description of all covariates tested
☐	☒	A description of any assumptions or corrections, such as tests of normality and adjustment for multiple comparisons
☐	☒	A full description of the statistical parameters including central tendency (e.g. means) or other basic estimates (e.g. regression coefficient) AND variation (e.g. standard deviation) or associated estimates of uncertainty (e.g. confidence intervals)
☐	☒	For null hypothesis testing, the test statistic (e.g. *F*, *t*, *r*) with confidence intervals, effect sizes, degrees of freedom and *P* value noted<br>*Give P values as exact values whenever suitable.*
☒	☐	For Bayesian analysis, information on the choice of priors and Markov chain Monte Carlo settings
☐	☒	For hierarchical and complex designs, identification of the appropriate level for tests and full reporting of outcomes
☐	☒	Estimates of effect sizes (e.g. Cohen's *d*, Pearson's *r*), indicating how they were calculated

Our web collection on statistics for biologists contains articles on many of the points above.

Software and code

Policy information about availability of computer code

Data collection	Methods and sources of data collection are stated in the text and supplemental methods.
Data analysis	Software used for data analysis included: GraphPad Prism (ver.7&8), R (ver.3.6.2), Limma (ver.3.40.6), RRHO (ver.1.24.0), WGCNA (ver.1.68), fgsea (ver.1.10.0), URA (ver. Fall 2019), ImageJ (ver.1.52p 22), STAR aligner (Ver.2.7), edgeR (ver.3.30.3)

For manuscripts utilizing custom algorithms or software that are central to the research but not yet described in published literature, software must be made available to editors and reviewers. We strongly encourage code deposition in a community repository (e.g. GitHub). See the Nature Research guidelines for submitting code & software for further information.

Data

Policy information about availability of data

All manuscripts must include a data availability statement. This statement should provide the following information, where applicable:

- Accession codes, unique identifiers, or web links for publicly available datasets
- A list of figures that have associated raw data
- A description of any restrictions on data availability

TRAP-seq data are available at GEO: GSE157021. Molecular Signatures Database (MSigDB) can be accessed here: www.gsea-msigdb.org/gsea/msigdb.

Field-specific reporting

Please select the one below that is the best fit for your research. If you are not sure, read the appropriate sections before making your selection.

[x] Life sciences [ ] Behavioural & social sciences [ ] Ecological, evolutionary & environmental sciences

For a reference copy of the document with all sections, see nature.com/documents/nr-reporting-summary-flat.pdf

Life sciences study design

All studies must disclose on these points even when the disclosure is negative.

Sample size	For behaviors, animal numbers were informed by previous experiments to inform power and effect size calculations, using the G*Power 3 software package. For translatomic studies, sample sizes were based on data from previous studies, see McCullough et. al., 2018
Data exclusions	For TRAP behavioral data, no samples were excluded based upon any criteria. For CREB validation experiments, one cage of animals was excluded from fear conditioning based upon wounding sustained due to an aggressive cage mate. In data analysis for TRAP data "Samples were retained according to the full capturing of their behavioral data and based on outlier determination, according to a consensus of hierarchical clustering, PCA and MDS. "
Replication	Translatomic analyses were verified using qPCR. All validation data is presented in the report.
Randomization	Mice of proper sex and genotype were randomized by a blinded experimenter.
Blinding	All behavioral experiments were completed by blinded experimenter. For fear conditioning, where blinding to group was not possible, cages assigned to each group were assigned by a blinded experimenter.

Reporting for specific materials, systems and methods

We require information from authors about some types of materials, experimental systems and methods used in many studies. Here, indicate whether each material, system or method listed is relevant to your study. If you are not sure if a list item applies to your research, read the appropriate section before selecting a response.

Materials & experimental systems

n/a	Involved in the study	
[x]	[ ]	Antibodies
[x]	[ ]	Eukaryotic cell lines
[x]	[ ]	Palaeontology and archaeology
[ ]	[x]	Animals and other organisms
[x]	[ ]	Human research participants
[x]	[ ]	Clinical data
[x]	[ ]	Dual use research of concern

Methods

n/a	Involved in the study	
[x]	[ ]	ChIP-seq
[x]	[ ]	Flow cytometry
[x]	[ ]	MRI-based neuroimaging

Animals and other organisms

Policy information about studies involving animals; ARRIVE guidelines recommended for reporting animal research

Laboratory animals	Mice. Both male and femal mice were used for initial TRAP discovery cohort. Male mice were used for qPCR validation and viral validation. All mice were 10 to 16 weeks old at time of experiment. C57BL/6J, B6;FVB-Tg(Crh-cre)1Kres/J, Rosa26 fs-TRAP; (B6.129S4-315 Gt(ROSA)26Sortm1(CAG-EGFP/Rpl10a,-birA)Wtp/J), B6(Cg)-Crhtm1(cre)Zjh/J.
Wild animals	*Provide details on animals observed in or captured in the field; report species, sex and age where possible. Describe how animals were caught and transported and what happened to captive animals after the study (if killed, explain why and describe method; if released, say where and when) OR state that the study did not involve wild animals.*
Field-collected samples	*For laboratory work with field-collected samples, describe all relevant parameters such as housing, maintenance, temperature, photoperiod and end-of-experiment protocol OR state that the study did not involve samples collected from the field.*
Ethics oversight	All procedures were approved by McLean Hospital Institutional Animal Care and Use Committee and complied with National institutes of Health guidelines.

Note that full information on the approval of the study protocol must also be provided in the manuscript.
